# A Snail Perspective on the Biogeography of Sulawesi, Indonesia: Origin and Intra-Island Dispersal of the Viviparous Freshwater Gastropod *Tylomelania*

**DOI:** 10.1371/journal.pone.0098917

**Published:** 2014-06-27

**Authors:** Thomas von Rintelen, Björn Stelbrink, Ristiyanti M. Marwoto, Matthias Glaubrecht

**Affiliations:** 1 Museum für Naturkunde - Leibniz Institute for Evolution and Biodiversity Science, Berlin, Germany; 2 Zoology Division (Museum Zoologicum Bogoriense), Research Center for Biology, LIPI, Cibinong, Bogor, Indonesia; University of Basel, Switzerland

## Abstract

The complex geological history of the Indonesian island Sulawesi has shaped the origin and subsequent diversification of its taxa. For the endemic freshwater snail *Tylomelania* a vicariant origin from the Australian margin has been hypothesized. Divergence time estimates from a mtDNA phylogeny based on a comprehensive island-wide sampling of *Tylomelania* fit regional tectonic constraints and support the ‘out-of-Australia’ vicariance hypothesis. The Banggai-Sula region of the Sula Spur, the Australian promontory colliding with West Sulawesi during the Miocene, is identified as a possible source area for the colonization of Sulawesi by the ancestor of *Tylomelania*. The molecular phylogeny also shows a rapid diversification of *Tylomelania* into eight major lineages with very little overlap in their distribution on the island. Haplotype networks provide further evidence for a strong spatial structure of genetic diversity in *Tylomelania*. Distribution boundaries of the major lineages do at best partially coincide with previously identified contact zones for other endemic species groups on Sulawesi. This pattern has likely been influenced by the poor dispersal capabilities and altitudinal distribution limits of this strict freshwater inhabitant. We suggest that late Miocene and Pliocene orogeny in large parts of Sulawesi has been the vicariant event driving primary diversification in *Tylomelania*.

## Introduction

The Indonesian island Sulawesi lies at the heart of the Indo-Australian Archipelago (IAA), one of the world's most species-rich areas and arguably the one with the most complex geography and geology [1]. The largely insular nature of the region sets it apart from the other major tropical rainforest areas [2]. While the entire IAA is essentially the product of continental collisions since the Paleozoic [3], the eastern part of the archipelago has its origin in the ongoing collision between Asia and Australia since the early Miocene [4]. An ancient continental core in the West that has been emergent since the Mesozoic – comprising Indochina and Sundaland – is thus sharply set apart from an area of predominantly young (Miocene to Pleistocene) oceanic islands including Sulawesi in the East bordered by the Australian continental margin [5]. The changes in the paleogeography of the IAA have left a strong signature in the distribution of the region's fauna and flora. Several faunal boundaries, including the famous ‘Wallace Line’ [6], were proposed to account for the observation that the western or eastern distribution limits of many animal groups are located within the oceanic island region of the IAA (see e.g., [7]). The transitional region Wallacea, which comprises all islands between the Sunda and Australian shelves, was proposed as one attempt to avoid the designation of a single faunal boundary [8]. While the concept of a transitional zone was quickly shown to be inadequate in itself [7], [9], the name Wallacea has stuck for the Indonesian part of this oceanic island assemblage [1], [10] and was also used to circumscribe one of SE Asia's four biodiversity hotspots [11], characterized by its unique, yet highly threatened biotic diversity. The fauna of the Wallacean islands is generally characteristic of oceanic islands [12], e.g., being “disharmonic”, i.e., depauperate at a higher taxonomic level, and having a high proportion of endemic species [9], [13].

Sulawesi stands out among the islands of Wallacea due to its size, different geological history and higher faunal diversity [14]. The geographic origin of Sulawesi's fauna and the mode of colonization of the island are still disputed to some degree even 150 years after the first proposal of the Wallace Line [15], [16]. One reason for the controversy lies in the fact that Sulawesi, in contrast to most of the other islands in Wallacea, is not truly oceanic, but a composite island at the centre of the Asia-Australia collision zone. Parts of the island were formerly attached to either the Asian or Australian continental margin and separated from these areas by vicariant processes (for details see [16]). In the West, the opening of the Makassar Strait separated West Sulawesi from Sundaland in the Eocene c. 45 Mya [4]. In the East, the traditional view of collisions of multiple micro-continental fragments sliced from New Guinea with an active volcanic margin in West Sulawesi at different times since the Early Miocene c. 20 Mya [17], [18] has recently been replaced by the assumption of extensional fragmentation following a single Miocene collision of the Sula Spur with West Sulawesi [4], [16], [19]. However, the revised geological hypothesis does not alter the theoretical possibility to invoke vicariance-based hypotheses for an origin of taxa on Sulawesi from either Asia or Australia [16].

The predominantly Asian origin of the island's biota has been recognized for a long time and has during the last two decades gained support from molecular phylogenetic studies involving taxa from across the Wallace line (see reviews by [1] and [20]). The recent dating of the colonization of Sulawesi in 20 non-marine animal groups by a molecular clock approach has shown that Miocene to Pleistocene dispersal to the island from Asia (Sundaland, Philippines) is the most likely mechanism for the origin of the vast majority of Sulawesi taxa [16]. However, an origin of Sulawesi taxa from both Asia and Australia (including New Guinea) through vicariant processes could not be ruled out in some instances, e.g., from Asia for mite harvestmen [21] or from Australia for pachychilid freshwater snails [22], atherinimorph fishes [23], and phalangerids [24].

Among the three taxa with an Australian origin, the pachychilid gastropods likely represent the strongest case for a vicariance scenario as the likelihood for dispersal in these strictly freshwater-dwelling animals seems extremely low. On Sulawesi, the group is solely represented by the endemic genus *Tylomelania* Sarasin & Sarasin, 1897 [25]. *Pseudopotamis* Martens, 1894, restricted to two of the North Australian Torres Strait islands, has been consistently identified as the sister group of *Tylomelania* [22], [26]–[28]. Both taxa are ovoviviparous and share the synapomorphy of a pallial oviduct modified into an uterine brood pouch releasing comparatively large and shelled juveniles [25]. Brooding in freshwater snails is generally regarded as being associated with a low dispersal potential [29], [30] which renders transoceanic dispersal across a distance of c. 2,000 km between Sulawesi and the Torres Strait Islands unlikely. This has prompted a hypothesis of vicariance through ‘terrane rafting’ from the north Australian margin [22], [25]. Again, the recent replacement of the concept of terrane rafting by the assumption of extensional fragmentation has not altered the basic premise of this hypothesis (see [16]). While the study of [16] has demonstrated that the estimated timing of the split between *Tylomelania* and *Pseudopotamis* is consistent with the geological data under the ‘out-of-Australia’ vicariance hypothesis, only a single sequence each from two species of *Tylomelania* was included.

As a result of its complex geological history (compare above), Sulawesi is geographically highly structured, primarily through its subdivision into the four arms constituting its k-shape. In addition, various mountain ranges, mostly resulting from the amalgamation of its constituent fragments in the Miocene [5], and some low-lying areas (e.g., Tempe and Gorontalo depressions) inundated during sea-level highstands act as potential barriers to dispersal across the island. This is reflected in the distribution patterns of species on Sulawesi, which exhibit strong geographic structuring in all taxa examined where sampling coverage across the island is sufficiently dense [31]–[37]. For some taxa, the phylogeographic breaks or contact zones, respectively, were found to be largely congruent, suggesting a strong role for habitat fragmentation in the diversification of Sulawesi taxa [31], [33]. For freshwater organisms, respective comparative data are still lacking, as most research has focused on the endemic aquatic radiations in the ancient lakes of Sulawesi (see review in [38]). *Tylomelania* should serve as a good model to trace the effect of historic vicariant barriers as the animals are not only restricted to freshwater, but their present-day distribution suggests that they are not capable of occurring at an altitude of more than about 700 m and they are only found near the coast where rivers or streams are directly descending from nearby mountains, or in karstic outcrops (pers. observation 1999–2011).

Consequently, we here study the phylogeography of *Tylomelania* based on samples from across its entire distribution range on Sulawesi to address questions pertaining to the origin of the taxon on Sulawesi and its subsequent diversification on the island. Specifically, we attempt to identify an area of origin on Sulawesi from the sequence of splits within *Tylomelania* and to link patterns of intra-island differentiation to Sulawesi's geology and topography. In addition, we discuss our data in comparison with those derived from other taxa that are widely distributed on Sulawesi, such as toads [31], [33], macaques [31], [39], fanged frogs [32], and tarsiers [40].

## Materials and Methods

### Ethics statement

Sampling at two localities in South Sulawesi within Bantimurung-Bulusaraung N.P. was done by RMM (co-author of this MS) from LIPI or N.P. staff, respectively, and given to the Research Center of Biology as the central biodiversity repository in Indonesia. The samples used in this study were given on loan and subsequently partly donated to Museum für Naturkunde by LIPI. All other sampling locations are outside of national parks or other protected areas and sampling required no permission in addition to the research permit(s) issued by LIPI or RISTEK as the responsible authorities in Indonesia. The field studies did not involve endangered or protected species. Detailed locality information is provided in Table S1.

### Material

**Figure 1:**
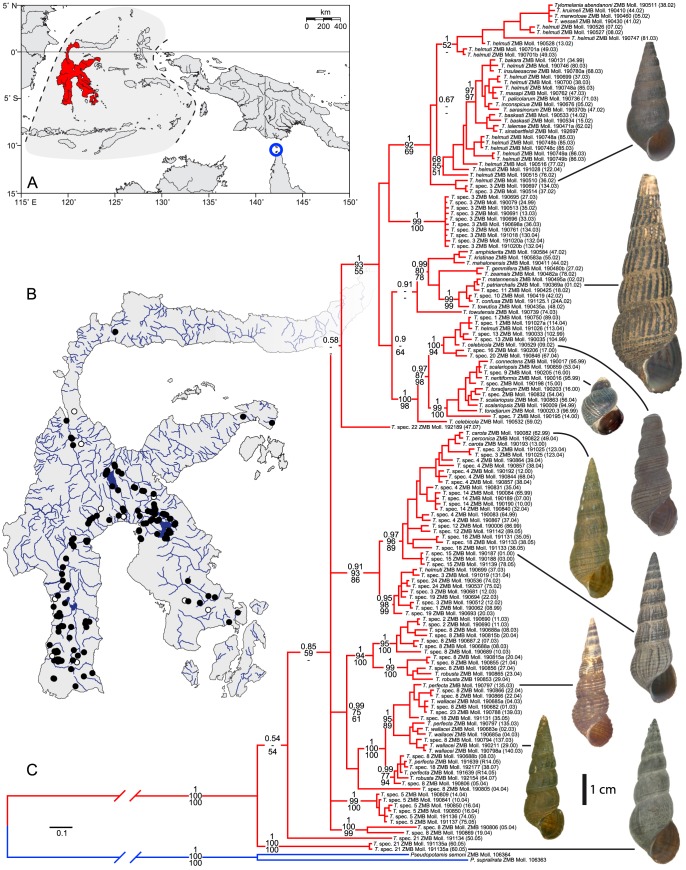
Distribution and molecular phylogeny of *Tylomelania*. A. Distribution area of *Tylomelania* (red) and its sister taxon *Pseudopotamis* (blue circle) on Sulawesi and the Torres Strait Islands, respectively. B. Map of Sulawesi with sample sites of *Tylomelania*. Black dot – sample sequenced for this study; white dot – museum sample. C. BI phylogram and shells of selected species of *Tylomelania*. Phylogeny based on 1,528 bp of mtDNA (COI and 16S). Numbers on branches show node support; BI posterior probability (top), ML (centre), and MP (bottom) bootstrap values. Colour code as in panel A.

Samples from 191 sites comprising 1,170 individuals of 62 species including 26 undescribed morphospecies (species delimitations are based on shell and radula characters that have been shown to be effective in distinguishing sympatric species in *Tylomelania* from the lakes of Sulawesi [41]) were collected across the entire distribution range of *Tylomelania* on Sulawesi (Figure 1, Table S1). Permits for conducting fieldwork were issued by LIPI (1999–2007) and RISTEK (from 2008) as the responsible authorities in Indonesia. All material has been preserved in 70–95% ethanol. Voucher specimens employed in this study are deposited in the Malacological Department and DNA samples are stored in the central DNA collection of the Museum of Natural History, Berlin (ZMB). Locality details and both museum and GenBank accession numbers for all sequenced animals are provided in Tables S1 & S2.

### Molecular methods

For the phylogeny, two mitochondrial gene fragments, a ∼890 bp region of the 16S ribosomal RNA gene (16S) and a 710 bp fragment (the so-called DNA barcoding fragment) of the Cytochrome Oxidase subunit I gene (COI) were sequenced in 160 specimens of *Tylomelania* using methods and primers described previously [42], [43]. The dataset was complemented by published sequences of both genes from 42 individuals [42]. Sequences of the two species of *Pseudopotamis* from the North Australian Torres Strait Islands were used as outgroup as suggested by previous studies [22], [26], [27]. COI was sequenced from an additional 968 specimens for the haplotype networks.

### Phylogenetic analyses

Orthologous DNA sequences were aligned by eye (COI) and with MUSCLE (16S; webserver) [44]. The MUSCLE alignment was corrected manually for unambiguous algorithm-specific errors. Following alignment, the 202 sequences obtained for each gene were reduced to unique haplotypes (Table S1) using DAMBE 5.066 [45]. The aligned sequence sets of COI (660 bp, 127 sequences; Figure S1) and 16S (868 bp, 136 sequences; Figure S2) were analyzed separately and combined into a single concatenated alignment of 157 sequences (1,528 bp; Figure 1).

Maximum Parsimony (MP) analyses were performed with PAUP*4.0b010 [46]; bootstrap replicates = 10,000; gaps were treated as fifth state). For Bayesian Inference (BI) analyses, the appropriate models of sequence evolution (HKY+G for COI and GTR+I+G for 16S) were determined using jModelTest 0.1.1 [47] both based on the Akaike Information Criterion and the Bayesian Information Criterion. The two genes were set as partitions in the concatenated dataset and analyses run with the model specified for each partition separately: ML with RAxML BlackBox [48] using GTR+G and 100 bootstrap replicates, and BI with MrBayes 3.1.2 [49]; ngen = 5,000,000 for single-gene datasets, and 10,000,000 for the combined dataset; samplefreq = 100/200; burnin = 35,001). For comparison, trees based on analysis of the separate datasets are provided as (Figures S1 and S2).

### Molecular clock analyses

A reduced COI dataset (N = 26) was tested for nucleotide substitution saturation using the test by [50] in DAMBE showing no significant saturation for COI. Strict and uncorrelated lognormal relaxed clock analyses were performed in BEAST v. 1.7.3 [51]; GTR+I+G; Yule process; ngen = 50,000,000; log = 1,000; burnin = 35,001) using a substitution rate of 1.76%/My as suggested by [52] for several freshwater taxa including gastropods. Fossils were not available for calibration. Log files of both runs were subjected to a Bayes factor analysis as implemented in Tracer 1.5 [53] resulting in a small value of 0.212 slightly supporting the strict clock analysis.

### Haplotype networks

The complete COI dataset (1,170 sequences) was reduced to 233 unique haplotypes (Figures S3 and S4, Table S2) using DnaSP v. 5.10.01 [54]. Haplotype networks were calculated from these haplotypes using TCS v. 1.2.1 [55] using the 95% parsimony criterion.

## Results

### Phylogeny of *Tylomelania*

**Figure 2:**
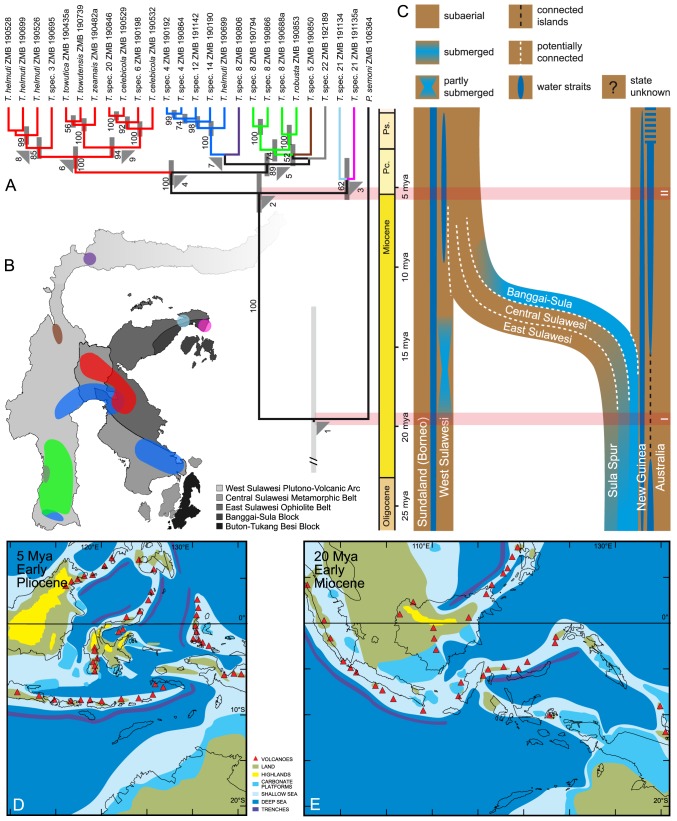
Calibrated phylogeny of *Tylomelania* and the tectonic history of Sulawesi. A. Bayesian (BEAST) chronogram of *Tylomelania* based on 660 bp of mtDNA (COI) using a substitution rate of 1.76%/My (see Material and methods). The major lineages of *Tylomelania* are colour-coded. The pink horizontal bars link the events associated with nodes 1 (TMRCA of *Tylomelania* and *Pseudopotamis*) and 2 (first speciation event within *Tylomelania*) with the geological timescale and paleogeography of Sulawesi (panel C). B. Map of Sulawesi with major tectonic subdivisions (compare panel C) and the distribution of the major lineages of *Tylomelania* (colour-coding of areas corresponds to major lineages in panel A). C. Schematic summary of the geographic connections and the timing of separation or collision of the different parts of Sulawesi (see map in panel B). Blue tinting indicates presumably submerged areas, see legend for details. Modified from [16]. D,E. Paleogeographic reconstructions of the Asia-Australia collision zone 5 Mya and 20 Mya. Modified from [56].

**Figure 3:**
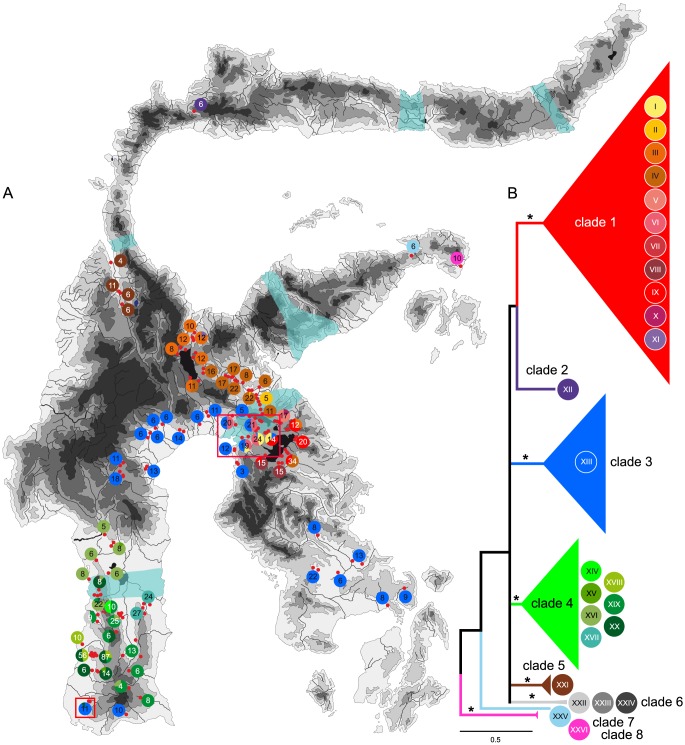
Distribution of the major lineages and haplotype groups of *Tylomelania* on Sulawesi. A. Sulawesi map with assignment of sampling sites (small red dots) to haplotype groups (pie charts, haplotype group specific colour code as in panel B). Haplotype group diversity for sites in close geographic vicinity has been subsumed within a single pie chart. Numbers within pie charts are sequenced individuals. Bluish areas indicate the position of contact zones for macaque species on Sulawesi (from [31]). Red frames show the position of the two contact zones of *Tylomelania* clades. B. BI phylogram based on tree shown in Figure 1, all clades with a BPP of >0.90 are shown as cartoons. Colours correspond to those used in Figure 2 and the circles with roman numbers indicate haplotype groups within each lineage (see Figure S1).

The molecular phylogeny based on MP, ML and BI analyses of the concatenated mtDNA dataset (COI and 16S) reveals eight major lineages (six clades and two distinct haplotypes) of *Tylomelania* on Sulawesi (Figures 1–3; Table 1). The monophyly of the six clades is supported by Bayesian Posterior Probabilities (BPP)>0.9, while only four have a MP and ML bootstrap support >80%. The splitting sequence of the eight lineages is not supported at all in the MrBayes topology (BPP<0.9; Figures 1 and 3) and only partially in the BEAST topology (Figure 2). Separate analyses for COI and 16S yielded essentially the same topology (Figures S1 and S2), differing only in the level of support for the major lineages. The eight major lineages are quite distinct, with an inter-lineage genetic distance range of 5.2–12.1% (COI) and 2.6–8.4% (16S), respectively (Table 2). A strong geographic pattern is evident in the distribution of the lineages of *Tylomelania*, with no to limited overlap in their distribution ranges (Figures 2 and 3). Three clades (1,3,4 – ‘red’, ‘blue’, and ‘green’ in Figures 2 and 3) are more widely distributed in East Central Sulawesi (clade 1), South-South West, South Central, and Southeast Sulawesi (clade 3), and Southwest Sulawesi (clade 4). All other lineages are confined to comparatively small regions.

**Table 1:** Intra- and inter-lineage genetic distances (p-distance) in *Tylomelania*.

	Clade 1 (0.0–9.1)	Clade 2 (-)	Clade 3 (0.2–4.4)	Clade 4 (0.2–7.6)	Clade 5 (0.2–0.8)	Clade 6 (3.6)	Clade 7 (-)	Clade 8 (-)
Clade 1 (0.0–5.7)	-	6.3–8.3	5.6–10.5	6.0–11.1	5.8–9.4	7.1–10.5	7.9–10.6	9.8–12.1
Clade 2 (-)	3.2–5.7	-	6.0–7.7	5.2–6.9	5.6–6.0	6.6	8.5	8.7
Clade 3 (0.0–4.3)	3.4–7.4	3.3–4.9	-	5.5–9.2	5.6–7.6	6.1–7.7	8.7–10.3	9.0–10.8
Clade 4 (0.0–4.1)	2.7–6.4	3.1–4.2	3.2–5.7	-	5.2–7.4	6.0–8.4	8.1–9.7	9.0–10.6
Clade 5 (0.1–0.2)	3.3–6.0	2.6–2.7	3.1–4.8	2.7–3.9	-	6.5–7.3	8.1–8.7	9.5–10.0
Clade 6 (3.0)	4.2–7.0	3.7–4.2	3.6–5.4	3.7–4.9	3.3–3.9	-	9.8–10.0	10.5–11.3
Clade 7 (-)	6.0–8.4	5.9	5.4–7.1	5.4–6.6	5.8–5.9	5.9–6.2	-	9.2
Clade 8 (0.1)	5.9–8.2	5.8–5.9	5.4–7.0	5.3–6.6	5.0–5.3	6.0–6.4	6.3–6.4	-

Top-right cells: COI; bottom-left cells: 16S. Intra-clade genetic distances are shown in brackets.

**Table 2:** Estimated maximum mean node ages from the strict clock analysis for COI (see Figure 2).

Node no.	Mean (My)	Lower 95% HPD	Upper 95% HPD
1	19.51	12.48	27.37
2	5.37	4.16	6.59
3	4.48	3.18	5.78
4	4.07	3.32	4.86
5	3.57	2.87	4.30
6	3.10	2.45	3.83
7	2.95	N.A.	N.A.
8	2.54	1.87	3.22
9	2.51	1.88	3.15

HPD – high posterior density.

The two basal lineages of *Tylomelania* (forming sister groups in the BEAST analysis, Figure 2) both occur on the easternmost Luwuk peninsula of the eastern arm of Sulawesi. The relationship of these two lineages to each other is not supported (BPP<0.7) in either BI analysis (Figures 1 and 2), though.

### Divergence time estimates

The results of the BEAST strict molecular clock analysis (Figure 2A, Table 2) suggest that the split between *Tylomelania* from Sulawesi and *Pseudopotamis* from the North Australian Torres Strait Islands occurred in the mid Miocene c. 19.5 Mya (Figure 2, Table 2, node 1) and the first diversification event on Sulawesi at the Miocene-Pliocene transition c. 5.4 Mya (Figure 2, Table 2, node 2). This basal split between the Luwuk peninsula lineage(s) and the rest of *Tylomelania* is followed by the subsequent diversification of the latter lineage into the major clades of *Tylomelania* in the mid to late Pliocene (c. 4-3 Mya).

### Spatial genetic diversity within *Tylomelania*

The TCS analyses yielded 26 distinct parsimony haplotype networks under a 95% confidence interval cut-off threshold (Table 3 and Table S1). Matching these haplotype networks to the major clades or lineages, respectively, obtained in the analysis of the concatenated dataset reveals a strong geographic structure in the genetic diversity of *Tylomelania* below the level of the major clades as well (Figure 3). In two areas in Southwest Sulawesi and in Central Sulawesi, two each of the three largest clades in terms of known distribution area (clade 1,3,4; red, blue and green in Figures 2 and 3) of *Tylomelania* overlap and haplotypes from both respective clades are found within a single population.

**Table 3:** Haplotype networks (groups) of *Tylomelania* with lineage assignment and intra-network genetic distance range (p-distance) for COI.

Lineage/clade	Haplotype network	N individuals	N haplotypes	COI
1				
	I	30	6	0.2–2.0
	II	7	3	0.6–0.8
	III	74	22	0.2–1.4
	IV	203	40	0.2–2.6
	V	22	4	0.2–0.6
	VI	50	9	0.2–0.6
	VII	12	1	-
	VIII	14	1	-
	IX	46	14	0.2–3.0
	X	3	1	-
	XI	1	1	-
2				
	XII	6	2	0.2
3				
	XIII	282	63	0.2–4.4
4				
	XIV	5	1	-
	XV	2	2	0.5
	XVI	56	7	0.2–1.7
	XVII	53	10	0.2–3.0
	XVIII	61	14	0.0–4.0
	XIX	82	5	0.5–1.1
	XX	111	18	0.0–3.7
5				
	XXI	27	4	0.2–0.8
6				
	XXII	5	1	-
	XXIII	2	1	-
	XXIV	1	1	-
7				
	XXV	6	2	0.3
8				
	XXVI	10	1	-

## Discussion

### The origin of *Tylomelania* on Sulawesi

The results of this study are compatible with the ‘out-of-Australia’ vicariance hypothesis for the origin of *Tylomelania* on Sulawesi [16], [22], [25]. The split of *Tylomelania* and *Pseudopotamis* (c. 19.5 Mya) matches or predates the separation of the Sula Spur from mainland New Guinea at 18-13 Mya (Figure 2, horizontal red bar marked ‘I’) and the first split within *Tylomelania* (c. 5.4 Mya) on Sulawesi matches or postdates the fusion of the different parts of Sulawesi at 10-6 Mya (Figure 2, horizontal red bar marked ‘II’).

Our results are largely in line with those from [16], who suggested an older age of 30.5 Mya (95% HPD: 42-20 Mya) for the split of *Tylomelania* and *Pseudopotamis* and a slightly younger age of c. 4.7 Mya (95% HPD: 7.8-1.9 Mya) for the first diversification event within *Tylomelania*. The congruence (considering the wide overlap in 95% HPD) between the results of both studies seems remarkable given that the dates from [16] were based on a much smaller sequence dataset for *Tylomelania* (from [27]) and a different marker (16S).

As pointed out by [16], there is a major conflict between the biological evidence as interpreted under the vicariance scenario and the distribution of land according to the geology-based paleogeographic reconstructions of the Sula Spur area during the Miocene (see e.g., Figure 2 and [5]). However, given the difficulties of recovering evidence for land in a region of small islands [56], we would follow [16] in arguing that the biological data might actually constitute harder evidence in this case. Under the vicariance scenario, the c. 15 My gap between the split of *Tylomelania* from *Pseudopotamis* and the first diversification event within *Tylomelania* could be explained by the restriction of the ancestral lineage of *Tylomelania* to a small and fluctuating distribution area on the Sula spur prior to the expansion of its distribution range on Sulawesi. These Sula Spur populations have most likely become extinct, as extensive searches on the Banggai (Banggai, Peleng; 2005, 2008) and Sula islands (Taliabu, Mangole, Sanana; 2008, 2013) were not successful.

One aim of this study has been to attempt to identify the region of Sulawesi that was initially colonized by the ancestors of *Tylomelania* by looking at the island-wide phylogeography of the taxon. The topology of the molecular phylogeny (Figures 1–3) suggests that the basal split within *Tylomelania* occurred between populations on the easternmost Luwuk peninsula and the rest of the island. The Luwuk peninsula is a fusion zone between East Sulawesi and the westernmost part of the Banggai-Sula fragment, both of which formed part of the Sula Spur (see Figure 2D,E). The amalgamation process of Sulawesi in the wake of the Sula Spur collision with West Sulawesi is poorly understood [5], but the Banggai-Sula fragment apparently became connected rather late in the late Miocene/early Pliocene to the core of present day Sulawesi, in contrast to parts of East Central and Southeast Sulawesi as borne out by evidence for land there since the Miocene [5]. Against this background, it is tempting to speculate that Sulawesi was colonized by the ancestor of *Tylomelania* from the Banggai-Sula area, particularly given the timing of the split between the lineages of *Tylomelania* from the Luwuk peninsula lineages and the remainder of the island, which at c. 5.4 Mya nicely matches the fusion of that part of the island to the rest of Sulawesi in the late Miocene-early Pliocene [57]. The topology of the BI tree as reconstructed with MrBayes (Figure 1) with a paraphyletic position of Luwuk peninsula *Tylomelania* relative to all other species would support this notion. However, the relationship of the Luwuk lineages to each other is not resolved and if the sister group relationship suggested in the BEAST analysis (Figure 2) should be correct rather than the topology derived from the MrBayes analyses (Figure 1), the assumption of an early isolation of a population on the eastern arm of Sulawesi would be an equally parsimonious alternative interpretation of that topology. Further progress on this issue will likely depend on filling the sampling gap between Central Sulawesi and the Luwuk peninsula on the Eastern peninsula.

### The geography of diversification on Sulawesi

The major lineages of *Tylomelania* on Sulawesi show a largely allo- or parapatric distribution (Figures 2 and 3), while with one exception each lineage has a continuous distribution area. The most widely distributed clade 3 (Figures 2 and 3, blue) occurs in three apparently disjunct areas in Southwest, Central and Southeast Sulawesi. The distribution of this clade overlaps with that of the other two widespread clades 1 and 4 (Figures 2 and 3, red and green) in South-Southwest Sulawesi and Central Sulawesi, respectively (Figure 3, red frames). The fact that haplotypes assigned to different clades are found among individuals within the same population (same sampling site within a 10–100 m stretch of stream or river, same morphospecies) suggests mitochondrial introgression in these contact zones. Given the distinctness of the mtDNA lineages involved (COI p-distance range between each of the three lineages: 5.6–11.1%), this is somewhat surprising. The lack of data from nuclear markers prevents an in-depth discussion at present, but the apparent absence of morphological hybrids might indicate that the introgression between the lineages is not a recent phenomenon. Different haplotypes are also found among individuals of a single and morphologically uniform population at the level of haplotype groups (separate networks under the 95% cut-off threshold) within the same clade (Figure 3). At the other extreme of the spectrum, the same haplotype can be shared among morphologically different and sympatrically occurring species in several localities (see e.g., haplotypes 1 or 2 from Southwest Sulawesi in Table S2). Without nuclear data, the cause for these phenomena – e.g., hybridization vs. incomplete lineage sorting (see e.g., [58] for possible causes of genetic discordance at the species level) – must remain speculative.

The strong geographic structure of genetic diversity in *Tylomelania* might be explained by various factors such as habitat fragmentation in the widest sense (e.g., vicariance through the rise of mountains or the formation of sea barriers), the development of ecological barriers, isolation by distance, or most likely a combination of these. If habitat fragmentation would be the predominant factor in shaping the distribution of the major lineages in *Tylomelania*, a strong correlation of lineage-specific distribution boundaries with geographic barriers irrespective of distance would be expected. While seemingly trivial, this concept can be hard to test, as today's barriers to dispersal may be very different from those in the past. This is particularly true for *Tylomelania* on Sulawesi, as the diversification into major lineages took place in the Pliocene and coincides with the onset of pronounced orogeny in many parts of Sulawesi [5]. The same is essentially true for ecological factors, such as climate, vegetation etc. However, *Tylomelania* has rather uniform ecological preferences with the notable exception of the species flocks in the ancient lakes of Sulawesi [38], [59], [60]. The homogenous morphology of the rasping tongue (radula), which is indicative of substrate in *Tylomelania*, reflects this [59], [60]. Thus, it does not seem very likely that ecological fragmentation has been a major factor in shaping distribution patterns. As geological vicariance will not just affect single taxa but a wide range of organisms, the search for congruent distribution patterns among unrelated taxa is a promising approach to identify such common barriers and a pivotal principle of comparative phylogeography and biogeography (see e.g., [61], [62]). On Sulawesi, the species or subspecies of many non-flying terrestrial and limnic taxa that were sampled across the island or larger parts are distributed parapatrically or allopatrically (see [31] for an overview). Molecular data are only available for a few widely sampled taxa from Sulawesi to date, though. Based on the spatial structure of genetic diversity in Celebes toads (*Bufo celebensis*) and macaques (*Macaca* spp.), [31] suggested that their contact zones were congruent and likely reflect habitat fragmentation by physical barriers.

While this interpretation of their data was questioned and isolation by distance proposed as an alternative hypothesis [63], coalescent analyses support the original habitat fragmentation scenario [33]. Similar patterns were observed in the fanged frog *Limnonectes* spp. [32], while *Chitaura* grasshoppers show at least partly deviant patterns [35] and *Lamprolepis* skinks show a very different North-South pattern [37]. The primary diversification events of these taxa took place from the earliest Pliocene (fanged frogs) through the middle Pliocene (macaques, grasshoppers) to the late Pliocene/Pleistocene (Celebes toads) [16]. Assuming that these estimates are correct (see [16] for some dating issues), the time of the primary speciation events on Sulawesi does not seem to play a major role in determining the congruence of contact zones or areas of endemism. The split into the major lineages of *Tylomelania* is hypothesized to have occurred in the mid Pliocene (Figure 2A) and does match the general timeframe of diversification of the taxa investigated so far. However, neither the distribution of the major lineages of *Tylomelania* nor of the constituent haplotype groups seems to be universally constrained by the barriers associated with the six macaque contact zones [31] (Figure 3). While the distribution boundaries of *Tylomelania* clades apparently match three of these, two other contact zones, across the north-central part of the Southwest peninsula and the northern part of the Southeast peninsula of the island, do not form a barrier for the three clades of *Tylomelania* (1,3,4) whose distribution extends across them. This suggests a partial fit of areas of endemism (at different taxonomic levels, though: species groups (lineages) in *Tylomelania* and species or subspecies in macaques or toads) of *Tylomelania* and the other taxa, for which an explanation might be sought through looking at potential differences in the nature (e.g., age) of the respective barriers. However, the sampling of *Tylomelania* is only sufficiently dense, i.e., including samples from the contact zone and both adjoining areas, to make a firm statement for the two contact zones at the southern arms of Sulawesi where these is a mismatch between areas of endemism. More samples will be needed to confirm that the three contact zones running across the eastern arm of Sulawesi, at the ‘neck’ just North of Central Sulawesi, and Gorontalo Depression (Figure 3) also form effective barriers for *Tylomelania*. Given that the timing of diversification is not fundamentally different in *Tylomelania*, other factors must play a role in causing any mismatch. *Tylomelania* is a strict freshwater dweller with apparent altitudinal distribution limits (see Introduction). This seems to be reflected in the distribution boundaries of the major clades, which are frequently formed by mountain ranges (Figure 3). The contact zones between clade 3 (Figures 2 and 3, blue) and clades 1 and 4 (Figures 2 and 3, red and green) do fit this hypothesis, as both contact zones are situated in areas where the topography is conducive to the – presumably secondary – contact of these clades. As a caveat, this interpretation is based on present-day topography. However, the orogeny of these mountain barriers most likely set in during the (early) Pliocene [5] and the bisection of formerly continuous drainages might have occurred practically instantly on a geological time scale.

A serious objection to a strict vicariant hypothesis correlated to orogeny arises from the obvious gaps in our data through insufficient sampling in some regions of Sulawesi, most notably in West Central Sulawesi and along the Eastern arm, but to a lesser degree also in Southeast Sulawesi and along the ‘neck’ of the island (*Tylomelania* does not occur in North Sulawesi East of the Gorontalo depression). The disjunct distribution of clade 3 (Figures 2 and 3, blue), e.g., is most likely a sampling artefact. At the same time and irrespective of sampling issues, the seemingly odd distribution of clade 3 around the Gulf of Bone could have an easy explanation through the recurrent Pleistocene sea-level drops, which increased the land area in the Gulf of Bone considerably [64].

To date, *Tylomelania* is the only freshwater taxon from Sulawesi with an island-wide sampling. It will be interesting to see whether our results are corroborated by ongoing studies on other freshwater taxa, such as several groups of gastropods, fishes, atyid shrimps and geocarcinid crabs (M. Glaubrecht, F. Herder, K. von Rintelen & C. Schubart, pers. comm.).

## Conclusions

Our molecular divergence time estimates are compatible with the tectonic framework for Sulawesi and thus provide further support for an ‘out-of-Australia’ vicariance scenario proposed for *Tylomelania* [16], [22], [25]. We also tentatively suggest that the ancestor of *Tylomelania* may have colonized the island from the Sula Spur region of Banggai-Sula, when its western part was fused to the rest of Sulawesi at the Miocene-Pliocene transition. The spatial distribution of genetic diversity as evidenced in *Tylomelania* does at best partially fit the pattern found in several other animal (terrestrial) groups such as macaques, toads or fanged frogs. The primary diversification of *Tylomelania* on Sulawesi into eight major lineages subsequent to the colonization of the island might have been shaped through vicariant events related to the orogeny of the island. Secondary contact between some clades is confined to two restricted areas and has resulted in mitochondrial introgression. These hypotheses could be tested by the future addition of nuclear markers and the sampling of crucial intermediate regions between the known distribution areas of the major lineages of *Tylomelania*.

## Supporting Information

Figure S1 Molecular phylogeny of *Tylomelania* based on COI only (BI phylogram); see text for details. Numbers on branches show node support; BI posterior probability (top), ML (centre), and MP (bottom) bootstrap values. Colours for clades correspond to those used in Figure 3.
(EPS)

Figure S2 Molecular phylogeny of *Tylomelania* based on 16S only (BI phylogram); see text for details. Numbers on branches show node support; BI posterior probability (top), ML (centre), and MP (bottom) bootstrap values. Colours for clades correspond to those used in Figure 3.
(EPS)

Figure S3 Haplotype networks for COI (part 1); see text for details. Colours for clades correspond to those used in Figure 3.
(EPS)

Figure S4 Haplotype networks for COI (part 2); see text for details. Colours for clades correspond to those used in Figure 3.
(EPS)

Table S1 List of sequenced specimens and sample provenience. The numbers listed under ZMB. Moll are the accession numbers of the malacological collection in the Museum für Naturkunde Berlin. This table lists all localities but only shows the accession numbers for sequences used for the tree reconstructions (Figures 1–3, S1, S2). Accession numbers for sequences that were only used for the computation of the haplotype networks (Fig. 3, S3, S4) are provided in Table S2, the respective samples are indicated by ‘HN’ in the COI sequence accession no. column. The letters in the haplotypes columns (A…Z, a…k) indicate specimens sharing the same haplotype, and bold type indicates the specimen shown in the tree(s). 1 = COI, 2 = 16S, 3 = concatenated dataset. All samples/sequences without an entry in the source column have been sequenced for this study.
(DOC)

Table S2 Haplotypes of *Tylomelania* (see Figures S3 & S4) and their assignment to species and museum vouchers. The numbers listed under ZMB Moll. are the museum accession numbers of the malacological collection of the Museum für Naturkunde Berlin and provide a link to the sample locality information in Table S1. GenBank accession numbers (for each haplotype) are for the specimen indicated in brackets.
(DOC)
