# Supplementary material for: A Snail Perspective on the Biogeography of Sulawesi, Indonesia: Origin and Intra-Island Dispersal of the Viviparous Freshwater Gastropod Tylomelania
Source: PLoS One. 2014 Jun 27;9(6):e98917. doi: 10.1371/journal.pone.0098917 (PMC4090239; doi:10.1371/journal.pone.0098917)
Supplement: Table S1 — List of sequenced specimens and sample provenience. The numbers listed under ZMB. Moll are the accession numbers of the malacological collection in the Museum für Naturkunde Berlin. This table lists all localities but only shows the accession numbers for sequences used for the tree reconstructions (Figures 1–3, S1, S2). Accession numbers for sequences that were only used for the computation of the haplotype networks (Fig. 3, S3, S4) are provided in Table S2, the respective samples are indicated by ‘HN’ in the COI sequence accession no. column. The letters in the haplotypes columns (A…Z, a…k) indicate specimens sharing the same haplotype, and bold type indicates the specimen shown in the tree(s). 1 = COI, 2 = 16S, 3 = concatenated dataset. All samples/sequences without an entry in the source column have been sequenced for this study. (DOC) [file pone.0098917.s005.doc]

Table S1. List of sequenced specimens and sample provenience. The numbers listed under ZMB. Moll are the accession numbers of the malacological collection in the Museum für Naturkunde Berlin. This table lists all localities but only shows the accession numbers for sequences used for the tree reconstructions (Figures 1-3, S1, S2). Accession numbers for sequences that were only used for the computation of the haplotype networks (Fig. 3, S3, S4) are provided in Table S2, the respective samples are indicated by ‘HN’ in the COI sequence accession no. column. The letters in the haplotypes columns (A…Z, a…k) indicate specimens sharing the same haplotype, and bold type indicates the specimen shown in the tree(s). 1 = COI, 2 = 16S, 3 = concatenated dataset. All samples/sequences without an entry in the source column have been sequenced for this study.

| **Taxon** | **ZMB Moll.** | **Locality data** | **GenBank accession no.** | | **Haplotypes** | | | **Source** |
| --- | --- | --- | --- | --- | --- | --- | --- | --- |
|  |  |  | **COI** | **16S** | **1** | **2** | **3** |  |
| *Pseudopotamis* |  | Australia: Queensland: Torres Strait Islands |  |  |  |  |  |  |
| *semoni* | 106363 | Hammond Island, creek on footpath to old gold mine; 10°33.04'S, 142°12.76'E | AY242967 | AY242968 |  |  |  | Glaubrecht & Rintelen 2003 |
| *supralirata* | 106364 | Prince of Wales Island, creek and pond at waterfall; 10°37.63'S, 142°11.53'E | AY242969 | AY242970 |  |  |  | Glaubrecht & Rintelen 2003 |
| *Tylomelania* |  | Indonesia: Sulawesi |  |  |  |  |  |  |
| *abendanoni* | 190511b | South Sulawesi: Lake Lontoa: W shore | KJ850747 | KJ850589 | **A** |  |  |  |
| *amphiderita* | 190584 | South Sulawesi: Lake Towuti: Loeha Island, W shore; 2°45.48'S, 121°30.9'E | KJ850748 | KJ850590 |  |  |  |  |
| *bakara* | 190131 | South Sulawesi: Lake Towuti: NW shore, Cape Bakara; 2°40.76'S, 121°26.09'E | AY311974 | AY311821 |  |  |  | Rintelen et al. 2004 |
| *baskasti* | 190533 | South Sulawesi: Larona catchment: Larona River; 2°40.36'S, 121°9.79'E | EF140859 | EF140843 |  |  |  | Rintelen et al. 2004 |
|  | 190534 | South Sulawesi: Larona catchment: Larona River; 2°40.55'S, 121°9.54'E | KJ850749 | EU881926 |  |  |  | this study / Rintelen & Glaubrecht 2008 |
| *carota* | 190082 | South Sulawesi: Kalaena catchment: Kalaena River, upper reaches; 2°26.2'S, 120°48.53'E | AY311958 | AY311823 | **B** |  |  | Rintelen et al. 2004 |
|  | 190193 | South Sulawesi: Kalaena catchment: Stream at road Wotu – Pendolo; 2°22.93'S, 120°47.9'E | AY311957 | AY311824 | B |  |  |  |
| *celebicola* | 190529 | Central Sulawesi: Tomori area: Lintumewure River, N of Nuha; 2°25.36'S, 121°21.43'E | KJ850750 | KJ850591 | **C** | **A** | **A** |  |
|  | 190530 | Central Sulawesi: Tomori area: Poaha River, N of Nuha; 2°22,055'S, 121°18,119'E | EF140864 | EF140848 | D | B | B | Rintelen et al. 2004 |
|  | 190531 | Sulawesi: Central Sulawesi: Tomori area: Lintumewure River, N of Nuha; 2°23.52'S, 121°19.66'E | KJ850751 | KJ850592 | C | A | A |  |
|  | 190532 | Central Sulawesi: Tomori area: Stream N of Nuha; 2°21.82'S, 121°18.05'E | EF140850 | EF140833 | **D** | **B** | **B** | Rintelen et al. 2004 |
|  |  |  | KJ850752 | KJ850593 | D | B | B |  |
|  | 191094a | Central Sulawesi: Tomori area: Stream at road Beteleme – Nuha; | HN | - |  |  |  |  |
|  | 191094b |  | HN | - |  |  |  |  |
|  | 191109 | Central Sulawesi: Tomori area: Stream at road Beteleme – Nuha; 2°13.52'S, 121°16.03'E | HN | - |  |  |  |  |
|  | 191110 | Central Sulawesi: Tomori area: Stream N of Wawopada, W of Beteleme; 2°09.05'S, 121°12.60'E | HN | - |  |  |  |  |
|  | 191607 | Central Sulawesi: Tomori area: Rumpi River, at road Nuha – Beteleme; 2°20.52'S, 121°18.06'E | HN | - |  |  |  |  |
|  | 191608a-c | Central Sulawesi: Tomori area: Stream at road Beteleme – Nuha; 2°20.20'S, 121°18.16'E | HN | - |  |  |  |  |
|  | 191609 | Central Sulawesi: Tomori area: Stream at road Beteleme – Nuha; 2°15.37'S, 121°16.86'E | HN | - |  |  |  |  |
|  | 191610 | Central Sulawesi: Tomori area: River in Tingkeao, at road Beteleme – Tomata; 2°08.52'S, 121°14.73'E | HN | - |  |  |  |  |
| *confusa* | 191125 | South Sulawesi: Lake Mahalona: SE shore, c. 30m W of Tominanga River outflow; 0°36,86'S, 121°30,9'E | KJ850753 | KJ850594 | **E** | **C** | **C** |  |
| *connectens* | 190017 | Central Sulawesi: Poso River: Sulewana rapids; 1°38,9'S, 120°39,26'E | AY311961 | AY311826 |  |  |  | Rintelen et al. 2004 |
| *gemmifera* | 190480b | South Sulawesi: Lake Matano: N shore; 2°29,049'S, 121°25,034'E | KJ850754 | KJ850595 |  |  |  |  |
| *helmuti* | 190209 | South Sulawesi: Towuti catchment: Stream at road Wawondula - Timampu; 2°38,46'S, 121°22,74'E | HN | - |  |  |  |  |
|  | 190510 |  | EF140851 | EF140834 |  |  |  | Rintelen et al. 2004 |
|  | 190515 | Southeast Sulawesi: Stream, just S of track Lengkobale - Parunela; 2°56,66'S, 121°30,81'E | EF140852 | EF140835 |  |  |  | Rintelen et al. 2004 |
|  | 190516 | Southeast Sulawesi: Tributary to Parudongka River, SE of Parunela; 2°56,74'S, 121°34,11'E | EF140853 | EF140836 | **F** |  |  |  |
|  | 190525 | South Sulawesi: Mahalona catchment: Tributary of Ponsoa River; 2°32,444'S, 121°33,345'E | HN | - |  |  |  |  |
|  | 190526 | South Sulawesi: Mahalona catchment: Tributary of Ponsoa River; 2°32,33'S, 121°32,23'E | EF140854 | EF140838 |  |  |  | Rintelen et al. 2004 |
|  | 190527 | South Sulawesi: Petea catchment: Tributary of Petea River; 2°31,8'S, 121°29,99'E | EF140855 | EF140839 |  |  |  | Rintelen et al. 2004 |
|  | 190528 | South Sulawesi: Petea catchment: Tributary of Petea River; 2°31,79'S, 121°28,81'E | KJ850755 | KJ850596 | **G** | **D** |  |  |
|  | 190699 | South Sulawesi: Larona catchment: Kondube River, at road N of Matompi, nr Timampu; 2°38,36'S, 121°24,98'E | KJ850756 | KJ850597 |  |  |  |  |
|  |  |  | KJ850757 | KJ850598 |  | **E** |  |  |
|  | 190700 | South Sulawesi: Larona catchment: Stream at road Wawondula - Timampu; 2°38,72'S, 121°23,48'E | KJ850758 | KJ85059 |  | E |  |  |
|  | 190701a | South Sulawesi: Towuti catchment: Bombongan River; 2°52,38'S, 121°22,54'E | KJ850759 | KJ850600 |  |  |  |  |
|  | 190701b |  | EF140856 | EF140840 | G |  |  | Rintelen et al. 2004 |
|  | 190746 | South Sulawesi: Tominanga catchment: N Tributary of Lampesue River; 2°35,61'S, 121°36,98'E | KJ850760 | KJ850601 |  |  |  |  |
|  | 190747 | South Sulawesi: Tominanga catchment: N tributary of Lampesue River; 2°35,47'S, 121°35,66'E | KJ850761 | KJ850602 | G | D |  |  |
|  | 190748a | South Sulawesi: Towuti catchment: Lemolemo River; 2°42,62'S, 121°40,99'E | KJ850762 | KJ850603 | **F** |  |  |  |
|  |  |  | KJ850763 | KJ850604 |  |  |  |  |
|  | 190748b |  | EF140857 | EF140841 | F |  |  | Rintelen et al. 2004 |
|  | 190748c |  | KJ850764 | KJ850605 | F |  |  |  |
|  | 190749a | South Sulawesi: Towuti catchment: Lengkona River; 2°40,82'S, 121°41,77'E | KJ850765 | KJ850606 | **H** |  |  |  |
|  | 190749b |  | EF140858 | EF140842 | H |  |  | Rintelen et al. 2004 |
|  | 191026 | South Sulawesi: Towuti catchment: Stream at road Beau - Masiku, tributary of Tokalimbu River; 2°50,732'S, 121°35,445'E | KJ850766 | KJ850607 |  | F |  |  |
|  | 191028 | South Sulawesi: Towuti catchment: Kakau River; 2°53,594'S, 121°31,458'E | KJ850767 | KJ850608 |  |  |  |  |
| *inconspicua* | 190676 | South Sulawesi: Lake Mahalona: SW shore; 2°36,64'S, 121°28,54'E | KJ850768 | KJ850609 |  |  |  |  |
| *insulaesacrae* | 190780a | South Sulawesi: Lake Towuti: N shore, bay E of Cape Bintu; 2°39,48'S, 121°33,25'E | KJ850769 | KJ850610 |  |  |  |  |
| *kristinae* | 190583a | South Sulawesi: Lake Towuti: Loeha Island, N shore; 2°45,67'S, 121°33,49'E | KJ850770 | KJ850611 |  |  |  |  |
| *kruimeli* | 190410 | South Sulawesi: Lake Mahalona: W shore; 2°35,88'S, 121°28,36'E | KJ850771 | KJ850612 | A |  |  |  |
| *lalemae* | 190471a | South Sulawesi: Lake Towuti: W shore, entrance of outlet bay, Cape Larona; 2°48,5'S, 121°25,04'E | KJ850772 | KJ850613 |  |  |  |  |
| *mahalonensis* | 190411 | South Sulawesi: Lake Mahalona: W shore; 2°35,88'S, 121°28,36'E | KJ850773 | KJ850614 |  |  |  |  |
| *masapensis* | 190762 | South Sulawesi: Lake Masapi: S shore; 2°50,84'S, 121°21,09'E | KJ850774 | KJ850615 |  |  |  |  |
| *marwotoae* | 190460 | South Sulawesi: Lake Mahalona: SW shore; 2°36,64'S, 121°28,54'E | KJ850775 | KJ850616 | A |  |  |  |
| *matannensis* | 190495a | South Sulawesi: Lake Matano: S shore; 2°30,2'S, 121°19,37'E | KJ850776 | KJ850617 |  | **G** |  |  |
| *neritiformis* | 190016 | Central Sulawesi: Poso River: Sulewana rapids; 1°38,9'S, 120°39,26'E | AY242959 | AY242960 |  | **H** |  | Rintelen et al. 2004 |
| *palicolarum* | 190736 | South Sulawesi: Lake Towuti: E shore, off Beau village; 2°48,99'S, 121°33,64'E | KJ850777 | KJ850618 |  |  |  |  |
| *patriarchalis* | 190369a | South Sulawesi: Lake Matano: S shore, W of islets; 2°28,462's, 121°15,584'E | KJ850778 | KJ850619 |  | G |  |  |
| *perconica* | 190822 | South Sulawesi: Kalaena catchment: Tributary of Tomoni River, W of Mangkutana; 2°29,207'S, 120°45,726'E | KJ850779 | KJ850620 | B |  |  |  |
| *perfecta* | 190797 | South Sulawesi: Maros carst: Bantimurung River, above waterfall; 5°0,96'S, 119°40,92'E | KJ850780 | KJ850621 |  |  |  |  |
|  |  |  | KJ850781 | KJ850622 |  |  |  |  |
|  | 191639 | South Sulawesi: 166m; 4°58,432'S, 120°1,8'E | KJ850782 | KJ850623 | **J** | **J** | **D** |  |
|  |  |  | KJ850783 | KJ850624 | **K** | J | **E** |  |
| *robusta* | 190811 | South Sulawesi: Langgara River (tributary of Dua River), Maroangin, off road Rappang - Enrekang; 3°44,414'S, 119°52,398'E | KJ850784 | KJ850625 | L | K | F |  |
|  | 190845 | South Sulawesi: Bilokka River, W of Bilokka, S of Amparita; 4°3,215'S, 119°49,1'E | KJ850785 | KJ850626 | L | K | F |  |
|  | 190853 | South Sulawesi: Tarrak River, S of Enrekang (=66-99); 3°41,762'S, 119°47,388'E | KJ850786 | KJ850627 |  |  |  |  |
|  | 190865 | South Sulawesi: Kupa, stream E of road Makassar - Parepare; 4°7,567'S, 119°37,426'E | KJ850787 | KJ850628 | **M** | **L** | **G** |  |
|  |  |  | KJ850788 | KJ850629 | M | L | G |  |
|  | 192152 | South Sulawesi: River at road Camba - Watampone; 4°53.139'S, 119°53.527'E | HN | - |  |  |  |  |
|  | 192154 | South Sulawesi: River at Kampungbaru, W-bound dead-end road off road Maros - Soppeng; 4°39,334'S, 119°53,503'E | KJ850789 | KJ850630 |  |  |  |  |
|  | 192155 | South Sulawesi: Rumpia River, S of road Pekkae - Soppeng; 4°35,891'S, 119°41,097'E | KJ850790 | KJ850631 | N | M | H |  |
|  | 193903 | South Sulawesi: Pucue River, Datae, road Parapare - Sidenreng; 3°54,893’S, 119°42,571’E | HN | - |  |  |  |  |
| *sarasinorum* | 190370b | South Sulawesi: Lake Towuti: Loeha Island, W shore; 2°45,48'S, 121°30,9'E | KJ850791 | KJ850632 |  |  |  |  |
| *scalariopsis* | 190008 | Central Sulawesi: Poso catchment: Saluopa waterfall; 1°44,95'S, 120°32,21'E | AY312007 | AY311892 | O | N | J | Rintelen et al. 2004 |
|  | 190009 | Central Sulawesi: Poso catchment: Stream c. 100m below Salopa waterfall; 1°44,9'S, 120°32,2'E | AY311962 | AY311831 |  |  |  | Rintelen et al. 2004 |
|  | 190859 | Central Sulawesi: Poso catchment: Creek in village, tributary of Poso River; 1°39,34'S, 120°39,957'E | KJ850792 | KJ850633 |  | H |  |  |
|  | 190863 | Central Sulawesi: Poso catchment: Saluopa waterfall; 1°44,95'S, 120°32,21'E | KJ850793 | KJ850634 | **O** | N | **J** |  |
|  | 191603 | Central Sulawesi: Poso catchment: Stream, N tributary of Lake Poso; 1°47.439'S, 120°30.799'E | HN | - |  |  |  |  |
| *sinabartfeldi* | 192697 | South Sulawesi: Larona catchment: Larona River, at road Malili - Soroako; 2°40,29'S, 121°9,81'E | KJ850794 | EU881924 |  |  |  | this study / Rintelen & Glaubrecht 2008 |
| spec. 1 | 190062 | South Sulawesi: Matano catchment: Stream at SW shore of Lake Matano, c. 200-500m above mouth; 2°27,77'S, 121°13,35'E | AY312005 | AY311888 |  | O |  | Rintelen et al. 2004 |
|  | 190750 | South Sulawesi: Towuti catchment: Tokalimbo River; 2°48,89'S, 121°34,89'E | KJ850795 | KJ850635 | **P** | **F** | **K** |  |
|  |  |  | KJ850796 | KJ850636 | P | F | K |  |
|  | 191027a | South Sulawesi: Towuti catchment: Masiku, Masiku River, tributary of Tokalimbu River; 2°51,988'S, 121°36,634'E | KJ850797 | KJ850637 |  | F |  |  |
|  | 191027b |  | HN | - |  |  |  |  |
| spec. 2 | 190690 | South Sulawesi: Bone carst: Large carstic resurgence, Lanca, at road Baleleng - Tokaseng; 4°23,031'S, 120°13,965'E | KJ850798 | KJ850638 | Q |  |  | Rintelen et al. 2004 |
|  |  |  | KJ850799 | KJ850639 | **Q** | **P** | **L** |  |
|  |  |  | KJ850800 | KJ850640 | Q | P | L |  |
| spec. 3 | 190079 | South Sulawesi: Larona catchment: Kondara River, at road Malili - Soroako; 2°35,17'S, 121°17,34'E | KJ850801 | KJ850641 | **R** | **Q** |  |  |
|  | 190512 | South Sulawesi: Matano catchment: Stream at S shore of Lake Matano; 2°28,6'S, 121°15,586'E | EF140861 | EF140845 |  | O |  | Rintelen et al. 2004 |
|  | 190513 | South Sulawesi: Matano catchment: Tapulemo River, W of Pontada, Soroako; 2°31,39'S, 121°20,22'E | KJ850802 | KJ850642 | R |  |  |  |
|  | 190514 | South Sulawesi: Larona catchment: Kondara River, nr Leduledu; 2°35,17'S, 121°17,3'E | KJ850803 | KJ850643 | **S** | **R** | **M** |  |
|  | 190681 | South Sulawesi: Malili drainage: Pongkeru River, at road Malili - Tolala; 2°41,895'S, 121°8,359'E | KJ850804 | KJ850644 |  |  |  |  |
|  | 190691 | South Sulawesi: Larona catchment: River, Balambano village, at road Malili - Soroako; 2°38,142'S, 121°12,856'E | KJ850805 | KJ850645 |  | **S** |  |  |
|  | 190692 | South Sulawesi: Larona catchment: Patingko River, at road to Tabarano, off road Malili - Soroako N of Balambano; 2°38,222'S, 121°14,56'E | EF140862 | EF140846 | S | R | M | Rintelen et al. 2004 |
|  | 190695 | South Sulawesi: Matano catchment: Stream at S shore of Lake Matano; 2°29,68'S, 121°18,4'E | KJ850806 | KJ850646 | **T** |  |  |  |
|  | 190696 | South Sulawesi: Larona catchment: Patingko River, S of road Malili - Soroako; 2°37,17'S, 121°14,95'E | KJ850807 | KJ850647 | R | S |  |  |
|  | 190697 | South Sulawesi: Larona catchment: Patingko River, at road Malili - Soroako, in Togo village (=24-99); 2°36,53'S, 121°15,56'E | KJ850808 | KJ850648 | S |  |  |  |
|  | 190698a | South Sulawesi: Larona catchment: Kondara River (tributary of Patingko River), at road Malili - Soroako, E of Leduledu; 2°35,11'S, 121°17,34'E | KJ850809 | KJ850649 | R |  |  |  |
|  | 190698b | South Sulawesi: Larona catchment: Kondara River (tributary of Patingko River), at road Malili - Soroako, E of Leduledu; 2°35,11'S, 121°17,34'E | KJ850810 | KJ850650 | S | R | M |  |
|  | 190761 | South Sulawesi: Matano catchment: Tapulemo River, W of Pontada, Soroako; 2°31,39'S, 121°20,22'E | EF140863 | EF140847 | **U** | Q | **N** | Rintelen et al. 2004 |
|  | 191018 | South Sulawesi: Kawata catchment: Kawata River, 50m W of road Ussu - Toletole, c. 2.5km S of Toletole; 2°33,008'S, 121°6,427'E | KJ850811 | KJ850651 | T | Q |  |  |
|  | 191019 | South Sulawesi: Cerekang catchment: Tributary of Wewu River, in Laroeha; 2°28,232'S, 121°4,122'E | KJ850812 | KJ850652 |  |  |  |  |
|  | 191020a | South Sulawesi: Kawata catchment: Kawata River, at road Ussu - Toletole, c. 0.7km S of Toletole; 2°31,95'S, 121°6,623'E | KJ850813 | KJ850653 |  | Q |  |  |
|  | 191020b |  | KJ850814 | KJ850654 | T |  |  |  |
|  | 191024 | South Sulawesi: Matano catchment: Soroako, stream W of Salonsa; 2°30,619'S, 121°19,785'E | KJ850815 | KJ850655 | U | Q | N |  |
|  | 191025 | South Sulawesi: Matano catchment: Soroako, stream W of Salonsa; 2°30,655'S, 121°19,671'E | KJ850816 | KJ850656 | **V** |  |  |  |
|  |  |  | KJ850817 | KJ850657 | V |  |  |  |
|  | 191119 | South Sulawesi: Larona catchment: Stream, tributary of Patingko River, nr Leduledu; 2°34.512'S, 121°16.524'E | HN | - |  |  |  |  |
|  | 191606 | South Sulawesi: Matano catchment: Soroako, Salonsa, tributary of Lake Matano, nr mouth (below 123-04); 2°30.375'S, 121°19.674'E | HN | - |  |  |  |  |
| spec. 4 | 190083 | South Sulawesi: Palopo plain: Stream along road Sabbang - Palopo, S of Sabbang; 2°36,68'S, 120°14,27'E | AY312001 | AY311884 |  |  |  | Rintelen et al. 2004 |
|  | 190191 | South Sulawesi: Palopo plain: Stream at road Palopo - Masamba; 2°39.98'S, 120°11.88'E | HN | - |  |  |  |  |
|  | 190192 | South Sulawesi: Palopo plain: Stream at road Palopo - Masamba; 2°34,36'S, 120°16,8'E | AY312000 | AY311883 |  |  |  | Rintelen et al. 2004 |
|  | 190831 | South Sulawesi: Palopo region: Malango River, Lebang, W of Palopo; 2°59,031'S, 120°9,879'E | KJ850818 | KJ850658 |  | **T** |  |  |
|  | 190844 | South Sulawesi: Palopo region: T. Nyarang River, btw Walenrang and Palopo; 2°57,756'S, 120°10,769'E | KJ850819 | KJ850659 |  |  |  |  |
|  | 190857 | South Sulawesi: Palopo plain: Kula River, Bone, N of Masamba; 2°32,635'S, 120°19,96'E | KJ850820 | KJ850660 |  |  |  |  |
|  |  |  | KJ850821 | KJ850661 |  |  |  |  |
|  | 190864 | South Sulawesi: Palopo plain: E of Patila, road Masamba - Wotu; 2°36,789'S, 120°34,383'E | KJ850822 | KJ850662 |  |  |  |  |
|  | 190867 | South Sulawesi: Palopo region: Latuppa, SW of Palopo; 3°1,515'S, 120°10,438'E | KJ850823 | KJ850663 |  |  |  |  |
|  | 192134 | South Sulawesi: Stream at road Masamba - Wotu; 2°34.356'S, 120°27.122'E | HN | - |  |  |  |  |
|  | 193899 | South Sulawesi: Palopo plain: Makawa River, tributary, road Palopo - Sabbang; 2°45.763'S, 120°8.503'E | HN | - |  |  |  |  |
|  | 193902 | South Sulawesi: Stream at road Masamba - Wotu; 2°34.356'S, 120°27.122'E | HN | - |  |  |  |  |
| spec. 5 | 190809 | Central Sulawesi: Palu valley: Sidaunta, at road Palu - Gimpu; 1°23,113'S, 119°58,563'E | KJ850824 | KJ850664 | **W** |  |  |  |
|  | 190841 | Central Sulawesi: Palu valley: Pakuli, at road Palu - Gimpu; 1°13,434'S, 119°56,861'E | KJ850825 | KJ850665 | W | U |  |  |
|  | 190850 | Central Sulawesi: Palu valley: Donggala Kecil, W of Palu; 0°53,943'S, 119°50,447'E | KJ850826 | KJ850666 | **X** |  |  |  |
|  |  |  | KJ850827 | KJ850667 | X | **U** |  |  |
|  | 191136 | Central Sulawesi: Palu valley: Ngangabomba River, S of Palu, Mantikole, Wera; 1°0,926'S, 119°51,72'E | KJ850828 | KJ850668 |  | U |  |  |
|  | 191137 | Central Sulawesi: Palu valley: Stream 15 km S of Palu, Mantikole; 1°1,174'S, 119°51,736'E | KJ850829 | KJ850669 |  | U |  |  |
| spec. 6 | 190198 | Central Sulawesi: Lake Poso: W shore, N of Bancea; 1°58,95'S, 121°35,11'E | AY311966 | AY311834 |  |  |  | Rintelen et al. 2004 |
| spec. 7 | 190195 | Central Sulawesi: Lake Poso: SW shore, E of Boe; 2°4,55'S, 120°39,45'E | AY311968 | AY311838 |  |  |  | Rintelen et al. 2004 |
| spec. 8 | 115517 | South Sulawesi: Maros carst: Assuloang Cave, main entrance; 4°55.746'S, 119°40.662'E | HN | - |  |  |  |  |
|  | 190682 | South Sulawesi: Maros carst: Tributary of Anda River, E of Celae, N of road; 4°55,216'S, 119°42,279'E | KJ850830 | KJ850670 | Y | **V** | **O** | Rintelen et al. 2004 |
|  | 190683d |  | KJ850831 | KJ850671 | Y | V | O |  |
|  | 190683g |  | KJ850832 | KJ850672 | Y | V | O |  |
|  | 190683b |  | HN | - |  |  |  |  |
|  | 190687 | South Sulawesi: Bone carst: Stream, nr Walesso, at road Tacippi - Uloe; 4°27,65'S, 120°11,18'E | KJ850833 | KJ850673 |  |  |  | Rintelen et al. 2004 |
|  | 190688a | South Sulawesi: Bone carst: Kacimpang River, in Watangulo, at road Mattirowalle - Baleleng; 4°24,385'S, 120°11,256'E | KJ850834 | KJ850674 | **Z** | **W** | **P** |  |
|  |  |  | KJ850835 | KJ850675 |  |  |  |  |
|  | 190688b |  | KJ850836 | KJ850676 | K |  |  |  |
|  |  |  | KJ850837 | KJ850677 | Z | J | E |  |
|  |  |  | KJ850838 | KJ850678 | K | W | P |  |
|  | 190689 | South Sulawesi: Bone carst: Public swimming pool at stream, E of Citta, 100m S of road Baleleng - Tokaseng; 4°22,565'S, 120°11,572'E | KJ850839 | KJ850679 |  |  |  |  |
|  | 190790 | South Sulawesi: Maros carst: Carstic resurgence, at cave entrance and river below; 4°54,17'S, 119°41,97'E | KJ850840 | KJ850680 | K | J | E | Rintelen et al. 2004 |
|  | 190793 | South Sulawesi: Maros carst: River at road N of Balocci; 4°54,63'S, 119°41,6'E | KJ850841 | KJ850681 | K | J | E | Rintelen et al. 2004 |
|  |  |  | KJ850842 | KJ850682 | K | J | E |  |
|  | 190794 | South Sulawesi: Maros carst: Carstic resurgence, in ricefields S of Balocci; 4°55,34'S, 119°40,95'E | KJ850843 | KJ850683 |  |  |  |  |
|  | 190796 | South Sulawesi: Maros carst; Small carstic resurgence, N of road Tonasa - Balocci; 4°53,48’S, 119°38,36’E | HN | - |  |  |  |  |
|  | 190805 | South Sulawesi: Carstic outflow, at road Pekkae - Ralla, W of Ralla; 4°32,354'S, 119°41,414'E | KJ850844 | KJ850684 |  |  |  |  |
|  | 190806 | South Sulawesi: River at road Pekkae - Ralla, btw Ralla and Kessi; 4°29,914'S, 119°44,087'E | KJ850845 | KJ850685 |  |  |  |  |
|  |  |  | KJ850846 | KJ850686 | **N** | **M** | **H** |  |
|  | 190807 | South Sulawesi: Stream at road Pekkae - Ralla, btw Ralla and Kessi; 4°29,926'S, 119°45,734'E | KJ850847 | KJ850687 | N | M | H |  |
|  | 190810 | South Sulawesi: Langkemma River, NW of Langkemma; 4°28,702'S, 119°52,682'E | KJ850848 | KJ850688 | K | J | E |  |
|  | 190815a | South Sulawesi: E of Campalawampang, road Takkalasi - Watansoppeng; 4°20,369'S, 119°41,418'E | KJ850849 | KJ850689 | **a** | **X** | **Q** |  |
|  |  |  | KJ850850 | KJ850690 | a | X | Q |  |
|  | 190815b |  | KJ850851 | KJ850691 |  |  |  |  |
|  |  |  | KJ850852 | KJ850692 | a | X | Q |  |
|  | 190855 | South Sulawesi: River at road Takkalasi - Watansoppeng, W of Watansoppeng; 4°20,199'S, 119°42,88'E | KJ850853 | KJ850693 | a |  |  |  |
|  | 190856 | South Sulawesi: Karajae River, Salokarajae, road Rappang - Enrekang; 3°46,653'S, 119°51,114'E | KJ850854 | KJ850694 | L | **K** | **F** |  |
|  | 190862 | South Sulawesi: Pucue River, Datae, road Parepare - Sidenreng; 3°54,893'S, 119°42,571'E | KJ850855 | KJ850695 | L | K | F |  |
|  |  |  | KJ850856 | KJ850696 | L | K | F |  |
|  | 190866 | South Sulawesi: Pacekke, E of Kirukiru (road E of Mangkoso); 4°15,705'S, 119°42,062'E | KJ850857 | KJ850697 |  | **Y** |  |  |
|  |  |  | KJ850858 | KJ850698 |  | Y |  |  |
|  | 190869 | South Sulawesi: E of Campalawampang, road Takkalasi - Watansoppeng; 4°19,919'S, 119°39,972'E | KJ850859 | KJ850699 | **b** | **Z** | **R** |  |
|  |  |  | KJ850860 | KJ850700 | b | Z | R |  |
|  | 191144a | South Sulawesi: River, tributary of Menraleng River, S of Takalala at road Takalala - Camba; 4°31.929'S, 119°57.784'E | HN | - |  |  |  |  |
|  | 191144b |  | HN | - |  |  |  |  |
|  | 191145 | South Sulawesi: Stream nr Woddi, W of road Takalala - Pekkae; 4°28.195'S, 119°54.518'E | HN | - |  |  |  |  |
|  | 191146 | South Sulawesi: Tributary of Labuajang River, at Langkemme, road Takalala - Pekkae; 4°29.587'S, 119°54.09'E | HN | - |  |  |  |  |
|  | 192146 | South Sulawesi: River at dead-end road off road Sinjai - Matango; 4°55.97'S, 120°7.638'E | HN | - |  |  |  |  |
|  | 192164 | South Sulawesi: Maros carst: River near karstic resurgence, N of Balocci; 4°54.152'S, 119°41.865'E | HN | - |  |  |  |  |
|  | 192171a | South Sulawesi: Maros carst: Karst river at dead-end road to Leang Londron; 4°51.929'S, 119°37.702'E | HN | - |  |  |  |  |
|  | 192171b |  | HN | - |  |  |  |  |
|  | 192183 | South Sulawesi: Maros carst: River at road Maros - Camba, in Battunuang; 5°3.083'S, 119°43.109'E | HN | - |  |  |  |  |
|  | 192188 | South Sulawesi: Maros carst: Stream N of road Maros - Camba; 5°2.569'S, 119°42.219'E | HN | - |  |  |  |  |
| spec. 9 | 190205 | Central Sulawesi: Lake Poso: S shore, Pendolo, at Hotel Mulia; 2°3,91'S, 120°41,5'E | KJ850861 | KJ850701 | **c** | H | **S** |  |
|  | 190861 | Central Sulawesi: Lake Poso: E shore, Cape Watulunto; 2°0,825'S, 120°42,007'E | KJ850862 | KJ850702 | c | H | S |  |
| spec. 10 | 190419 | South Sulawesi: Petea River: c. 1km from mouth at Lake Mahalona; 2°34,27'S, 121°31,07'E | KJ850863 | KJ850703 |  | C |  |  |
| spec. 11 | 190425 | South Sulawesi: Petea River: c. 2 km from Lake Matano outflow; 2°32,67'S, 121°29,48'E | KJ850864 | KJ850704 |  |  |  |  |
| spec. 12 | 190006 | Southeast Sulawesi: Moramo waterfalls; 4°13,24'S, 122°44,66'E | AY312002 | AY311891 |  |  |  | Rintelen et al. 2004 |
|  | 191142 | Southeast Sulawesi: Stream at Panganjaya. Road SE of Punggaluku; 4°23,284'S, 122°35,553'E | KJ850865 | KJ850705 |  |  |  |  |
|  | 191143 | Southeast Sulawesi: Moramo waterfalls; 4°13.24'S, 122°44.66'E | HN | - |  |  |  |  |
| spec. 13 | 190026 | Central Sulawesi: Tomori area: Mawaro River at road Tomata - Beteleme; 2°0.25'S, 121°0.48'E | HN | - |  |  |  |  |
|  | 190030 | Central Sulawesi: Tomori area: River at road Tomata - Beteleme; 2°1.16'S, 121°1.94'E | HN | - |  |  |  |  |
|  | 190031 | Central Sulawesi: Tomori area: Matang River at Road Tomata - Beteleme; 2°5.93'S, 121°7.28'E | HN | - |  |  |  |  |
|  | 190033 | Central Sulawesi: Tomori area: Puawu River, waterfall at road Tomata - Beteleme; 2°5,93'S, 121°9,82'E | AY312012 | AY311897 |  |  |  | Rintelen et al. 2004 |
|  | 190035 | Central Sulawesi: Tomori area: Stream at road Tampira - Beteleme; 2°7,11'S, 121°20,16'E | AY312013 | AY311898 |  |  |  |  |
|  | 191092a | Central Sulawesi: Tomori area: Ensa River, at road Tomata - Beteleme; 2°1.084'S, 121°4.321'E | HN | - |  |  |  |  |
|  | 191092b |  | HN | - |  |  |  |  |
|  | 191092c |  | HN | - |  |  |  |  |
|  | 191093 | Central Sulawesi: Tomori area: Ampere River, at road Tomata - Beteleme; 2°1.709'S, 121°5.38'E | HN | - |  |  |  |  |
|  | 191112a | Central Sulawesi: Tomori area: River at road Tomata - Beteleme; 2°5.908'S, 121°7.273'E | HN | - |  |  |  |  |
|  | 191112b |  | HN | - |  |  |  |  |
|  | 191114 | Central Sulawesi: Tomori area: Stream at road Tentena - Tomata (=98-99); 1°48.62'S, 120°46.43'E | HN | - |  |  |  |  |
|  | 191115 | Central Sulawesi: Tomori area: River at road Tomata - Beteleme (=99-99); 2°0.305'S, 121°0.456'E | HN | - |  |  |  |  |
|  | 191116a | Central Sulawesi: Tomori area: River at road Tomata - Beteleme; 2°0.882'S, 121°2.019'E | HN | - |  |  |  |  |
|  | 191116b |  | HN | - |  |  |  |  |
|  | 191118a | Central Sulawesi: Tomori area: Stream at road Tomata - Beteleme; 2°1.059'S, 121°3.925'E | HN | - |  |  |  |  |
|  | 191118b |  | HN | - |  |  |  |  |
|  | 191120 | Central Sulawesi: Tomori area: Stream, tributary of Puawu River, at road Tomata - Beteleme; 2°5.972'S, 121°9.672'E | HN | - |  |  |  |  |
|  | 191121 | Central Sulawesi: Tomori area: River at road Tomata - Beteleme; 2°4.911'S, 121°6.668'E | HN | - |  |  |  |  |
|  | 191611a | Central Sulawesi: Tomori area: Puawu River, cascades, at road Beteleme - Tomata, W of Wawopada; 2°9.314'S, 121°12.042'E | HN | - |  |  |  |  |
|  | 191611b |  | HN | - |  |  |  |  |
|  | 191613a | Central Sulawesi: Tomori area: Puawu River, at road Beteleme - Tomata; 2°5.984'S, 121°7.325'E | HN | - |  |  |  |  |
|  | 191613b |  | HN | - |  |  |  |  |
|  | 192158 | Central Sulawesi: Tomori area: Small river at road Taripa - Pendolo; 1°55.157'S, 120°47.697'E | HN | - |  |  |  |  |
|  | 192160 | Central Sulawesi: Tomori area: River at road Taripa - Pendolo; 1°59.163'S, 120°46.5'E | HN | - |  |  |  |  |
| spec. 14 | 190084 | South Sulawesi: Toraja land: Dolok River, at road Rantepao - Enrekang, N of Belajen; 3°18,14'S, 119°49,35'E | AY312017 | AY311901 |  | T |  |  |
|  | 190189 | South Sulawesi: Toraja land: Rantepao, Marante River; 2°57,42'S, 119°55,78'E | AY312015 | AY311899 | **d** |  |  | Rintelen et al. 2004 |
|  | 190190 | South Sulawesi: Toraja land: Tributary of Kambuna River, off road Rantepao - Palopo; 2°54,97'S, 119°59,26'E | AY312016 | AY311900 |  |  |  | Rintelen et al. 2004 |
|  | 190840 | South Sulawesi: Toraja land: Pakampan (tributary of Saa River, Noling drainage); 3°4,169'S, 119°58,638'E | KJ850866 | KJ850706 | d | T | **T** |  |
|  | 190851 | South Sulawesi: Toraja land: Marara River, W of Ledo (Sadang drainage); 3°1,993'S, 119°56,409'E | KJ850867 | KJ850707 | d | T | T |  |
|  | 190868 | South Sulawesi: Toraja land: Lengko River (Noling drainage), N of Simparrun; 3°4,887'S, 119°58,738'E | KJ850868 | KJ850708 | d | T | T |  |
|  | 191593a | South Sulawesi: Toraja land: Natural swimming pool "Tilangga"; 3°2.129'S, 119°53.233'E | HN | - |  |  |  |  |
|  | 191593b |  | HN | - |  |  |  |  |
|  | 191594 | South Sulawesi: Toraja land: Sa'dan River, at road Rantepao - Makale; 3°3.595'S, 119°52.07'E | HN | - |  |  |  |  |
|  | 191595 | South Sulawesi: Toraja land: Tributary of Rembon River and small inflowing stream, at road Makale - Mamasa; 3°0.478'S, 119°42.648'E | HN | - |  |  |  |  |
|  | 191596 | South Sulawesi: Toraja land: Stream at road Makale - Mamasa; 3°1.748'S, 119°44.318'E | HN | - |  |  |  |  |
| spec. 15 | 190187 | Southeast Sulawesi: Amberewa River, at road Kolaka - Kendari; 3°58,03'S, 122°15,77'E | AY312003 | AY311885 | **e** |  |  | Rintelen et al. 2004 |
|  | 190188 | Southeast Sulawesi: Watudehoa River, at road Lambuya - Aopa; 4°2,96'S, 122°6,86'E | AY312004 | AY311886 | e |  |  |  |
|  | 191138 | Southeast Sulawesi: Stream in Labela, E of Pundidaha, road Kendari - Kolaka; 3°57.897'S, 122°20.739'E | HN | - |  |  |  |  |
|  | 191139 | Southeast Sulawesi: Humbuti River, NW of Abuki; 3°38,91'S, 121°52,38'E | KJ850869 | KJ850709 | e |  |  |  |
|  | 191140 | Southeast Sulawesi: Simbune River, c. 1km NE of Raterate, road Kendari - Kolaka; 4°2.326'S, 121°54.204'E | HN | - |  |  |  |  |
|  | 191141a | Southeast Sulawesi: Poniponiki River, NW of Raterate, road Kendari - Kolaka; 4°2.115'S, 121°52.816'E | HN | - |  |  |  |  |
|  | 191141b |  | HN | - |  |  |  |  |
| spec. 16 | 190206 | Central Sulawesi: Poso catchment: Stream at road Pendolo - Bancea; 2°2,37'S, 120°37,25'E | AY312009 | AY311887 |  |  |  | Rintelen et al. 2004 |
| spec. 17 | 190812 | Central Sulawesi: Poso River: Sulewana, rapids; 1°38,871'S, 120°39,279'E | KJ850870 | KJ850710 | c | H | S |  |
| spec. 18 | 191131 | South Sulawesi: Creek running from NE into Pamukulu River at road crossing; 5°24,422'S, 119°36,722'E | KJ850871 | KJ850711 |  |  |  |  |
|  |  |  | KJ850872 | KJ850712 |  |  |  |  |
|  | 191132 | South Sulawesi: Creek running into Pamukulu River, further W, tributary down to river; 5°24,116’S, 119°36,24’E | HN | - |  |  |  |  |
|  | 191133 | South Sulawesi: Eremerasa swimming pool, N of Bantaeng; 5°28,896'S, 120°0,313'E | KJ850873 | KJ850713 |  |  |  |  |
|  |  |  | KJ850874 | KJ850714 |  |  |  |  |
|  | 192143 | South Sulawesi: River at road Bulukumba - Sinjai; 5°19,659'S, 120°7,97'E | KJ850875 | KJ850715 | J | J | D |  |
|  | 192177 | South Sulawesi: Malawa spring, near Patuku; 5°3,512'S, 119°57,475'E | KJ850876 | KJ850716 | J |  |  |  |
|  | 193900 | South Sulawesi: River at road Sinjai - Manipi, c. 2 km before Manipi; 5°13.2'S, 120°0.49'E | HN | - |  |  |  |  |
|  | 193901 | South Sulawesi: Aparang River, at Road Bulukumba - Sinjai, N of Bikeru; 5°15.13'S, 119°10.14'E | HN | - |  |  |  |  |
| spec. 19 | 190693 | Southeast Sulawesi: Tolala Region: Tolala River, E of Tolala at road to Loka; 2°56,05'S, 121°6,96'E | KJ850877 | KJ850717 |  |  |  |  |
|  | 190694 | Southeast Sulawesi: Tolala Region: River at road Malili - Tolala; 2°55,21'S, 121°5,7'E | EF140865 | EF140849 |  |  |  | Rintelen et al. 2004 |
|  | 190839 | Southeast Sulawesi: Tolala Region: River at road Malili - Tolala; 2°55,21'S, 121°5,7'E | HN | - |  |  |  |  |
| spec. 20 | 190207 | Central Sulawesi: Poso catchment: Stream at road Bancea - Tentena; 1°49.01'S, 120°31.03'E | HN | - |  |  |  |  |
|  | 190832 | Central Sulawesi: Poso catchment: Creek, tributary of Poso River; 1°41,019'S, 120°39,496'E | KJ850878 | KJ850718 |  |  |  |  |
|  | 190846 | Central Sulawesi: Poso catchment: Salokuwa River, tributary of Kodina River, S of Pendolo; 2°9.399'S, 120°43.943'E | KJ850879 | KJ850719 |  |  |  |  |
|  | 191597 | Central Sulawesi: Poso catchment: Stream at road Tentena - Peura, E shore of Lake Poso; 1°49.983'S, 120°38.242'E | HN | - |  |  |  |  |
|  | 191598 | Central Sulawesi: Poso catchment: Small stream, road Tentena - Toaro; 1°44.079'S, 120°40.098'E | HN | - |  |  |  |  |
|  | 191599 | Central Sulawesi: Poso catchment: Small stream, road Tentena - Toaro; 1°44.234'S, 120°40.039'E | HN | - |  |  |  |  |
|  | 191600 | Central Sulawesi: Poso catchment: Kelei, c. 5km SE of Tentena, small stream, tributary of Wimbi River; 1°47.5'S, 120°41.413' | HN | - |  |  |  |  |
|  | 191601 | Central Sulawesi: Poso catchment: Tributary of Kaiuku River, c. 30m from river, at road Tentena - Pendolo; 1°46.29'S, 120°42.98'E | HN | - |  |  |  |  |
|  | 191602 | Central Sulawesi: Poso catchment: Stream at road Poso - Tentena; 1°37.48'S, 120°42.131'E | HN | - |  |  |  |  |
|  | 191604 | Central Sulawesi: Poso catchment: Tonusu, c. 3km S of Tonusu towards Siuri; 1°48.95'S, 120°31.18'E | HN | - |  |  |  |  |
|  | 191605 | Central Sulawesi: Poso catchment: Uebangke River, N tributary of Lake Poso; 1°46.48'S, 120°35.61'E | HN | - |  |  |  |  |
| spec. 21 | 191134 | Central Sulawesi: Luwuk Peninsula: Biak River, above cascades, at road Luwuk - Pagimana, S of Salodik; 0°50,242'S, 122°52,394'E | KJ850880 | KJ850720 | **f** | **a** | **U** |  |
|  |  |  | KJ850881 | KJ850721 | f | a | U |  |
|  | 191135a | Central Sulawesi: Luwuk Peninsula: Stream in mountains nr Sape, SW of Balantak, E coast of Balantak Peninsula; 0°56,682'S, 123°21,38'E | KJ850882 | KJ850722 | **g** |  |  |  |
|  |  |  | KJ850883 | KJ850723 | g |  |  |  |
| spec. 22 | 192189 | Central Sulawesi: River at road Kotaraya - Tolitoli; 0°39,807'N, 120°39,409'E | KJ850884 | KJ850724 | **h** | **b** | **V** |  |
|  |  |  | KJ850885 | KJ850725 | h | b | V |  |
| spec. 23 | 190788 | South Sulawesi: Maros carst: Carstic resurgence, at cave entrance and river below; 4°54,17'S, 119°41,97'E | KJ850886 | KJ850726 | **Y** | **c** | **W** |  |
|  |  |  | KJ850887 | KJ850727 | Y | c | W |  |
|  | 190791 | South Sulawesi: Maros carst: River at road N of Balocci; 4°54,63'S, 119°41,6'E | KJ850888 | KJ850728 | Y | c | W |  |
|  | 192149 | South Sulawesi: Maros carst: Karstic river at mouth of Leang Londron cave; 4°51.746'S, 119°38.047'E | HN | - |  |  |  |  |
|  | 192162 | South Sulawesi: Maros carst: River near karstic resurgence, N of Balocci; 4°54.152'S, 119°41.865'E | HN | - |  |  |  |  |
|  | 192173 | South Sulawesi: Maros carst: Karst river at dead-end road to Leang Londron; 4°51.929'S, 119°37.702'E | HN | - |  |  |  |  |
| spec. 24 | 190536 | Southeast Sulawesi: Malili catchment: Stream at road Malili - Tolala; 2°49,77'S, 121°3,67'E | EF140860 | EF140844 |  | **d** |  | Rintelen et al. 2004 |
|  | 190537 | Southeast Sulawesi: Malili catchment: Stream at road Malili - Tolala; 2°48,9'S, 121°3,62'E | KJ850889 | KJ850729 |  | d |  |  |
| spec. 25 | 190032 | Central Sulawesi: Tomori area: Matang River at Road Tomata - Beteleme; 2°5.93'S, 121°7.28'E | HN | - |  |  |  |  |
|  | 191113 | Central Sulawesi: Tomori area: River at road Tomata - Beteleme; 2°5.908'S, 121°7.273'E | HN | - |  |  |  |  |
|  | 191612 | Central Sulawesi: Tomori area: Puawu River, at road Beteleme - Tomata; 2°5.984'S, 121°7.325'E | HN | - |  |  |  |  |
| spec. 26 | 195096 | South Sulawesi: Maros carst: Sambueja Cave, river in front of cave; 5°3.75'S, 119°41.28'E | HN | - |  |  |  |  |
| *toradjarum* | 190020 | Central Sulawesi: Lake Poso: Tentena, E shore, near outflow of Poso River; 1°45,92'S, 120°38,42'E | AY242961 | AY242962 |  |  |  | Rintelen et al. 2004 |
|  | 190203 | Central Sulawesi: Lake Poso: S shore, Pendolo, at Hotel Mulia; 2°3,91'S, 120°41,5'E | AY311967 | AY311836 |  |  |  | Rintelen et al. 2004 |
| *towutensis* | 190739 | South Sulawesi: Lake Towuti: S shore, c. 2 km E of Cape Mea; 2°55,8'S, 121°26,92'E | KJ850890 | KJ850730 |  |  |  |  |
| *towutica* | 190435a | South Sulawesi: Lake Towuti: Loeha Island, W shore; 2°46,18'S, 121°31,3'E | KJ850891 | KJ850731 |  |  |  |  |
| *wallacei* | 115515 | South Sulawesi: Maros carst: Rumbia Cave, pond in front of cave outflow; 5°2.185'S, 119°37.683'E | HN | - |  |  |  |  |
|  | 115516 | South Sulawesi: Maros carst: small stream below gallery entrance of Gua Assuloang; 4°55.579'S, 119°40.716'E | HN | - |  |  |  |  |
|  | 190211 | South Sulawesi: Lejang, public pool "Mattampa", at road Pare-Pare - Makassar, N of Pangkajene; 4°48,38'S, 119°32,66'E | AY312006 | AY311903 | **j** |  |  |  |
|  | 190683e | South Sulawesi: Maros carst: Tributary of Anda River, E of Celae, N of road; 4°55,216'S, 119°42,279'E | KJ850892 | KJ850732 |  |  |  |  |
|  | 190683a,c,f |  | HN | - |  |  |  |  |
|  | 190685a | South Sulawesi: Maros carst: Anda River, W of Celae; 4°54,728'S, 119°40,632'E | KJ850893 | KJ850733 | **k** | **e** | **X** |  |
|  |  |  | KJ850894 | KJ850734 | Y | **f** | **Y** |  |
|  | 190685b |  | KJ850895 | KJ850735 | k | e | X | Rintelen et al. 2004 |
|  | 190685c |  | KJ850896 | KJ850736 | Y | f | W |  |
|  |  |  | KJ850897 | KJ850737 | Y | c | Y |  |
|  | 190686 |  | HN | - |  |  |  |  |
|  | 190789 | South Sulawesi: Maros carst: Carstic resurgence, at cave entrance and river below; 4°54,17'S, 119°41,97'E | KJ850898 | KJ850738 | Y | V | O |  |
|  | 190792 | South Sulawesi: Maros carst: River at road N of Balocci; 4°54,63'S, 119°41,6'E | KJ850899 | KJ850739 | Y | c | W | Rintelen et al. 2004 |
|  |  |  | KJ850900 | KJ850740 | K | J | E |  |
|  | 190795 | South Sulawesi: Maros carst: Small carstic resurgence, N of road Tonasa - Balocci; 4°53,48'S, 119°38,36'E | KJ850901 | KJ850741 | Y | c | W |  |
|  | 190798a | South Sulawesi: Maros carst: Lejang, public pool "Mattampa", at road Pare-Pare - Makassar, N of Pangkajene (=29-00); 4°48,38'S, 119°32,66'E | KJ850902 | KJ850742 | j | **g** | **Z** |  |
|  | 190798b |  | KJ850903 | KJ850743 | j | g | Z |  |
|  | 192148 | Sulawesi: South Sulawesi: River at road Sinjai - Matango; 4°45.973'S, 120°4.357'E | HN | - |  |  |  |  |
|  | 192150 | South Sulawesi: Maros carst: Karstic river at mouth of Leang Londron cave; 4°51.746'S, 119°38.047'E | HN | - |  |  |  |  |
|  | 192163 | South Sulawesi: Maros carst: River near karstic resurgence, N of Balocci; 4°54.152'S, 119°41.865'E | HN | - |  |  |  |  |
|  | 192167a,b | South Sulawesi: Maros carst: Concrete canal at dead-end road to Leang Londron; 4°52.259'S, 119°37.508'E | HN | - |  |  |  |  |
|  | 192172a,b | South Sulawesi: Maros carst: Karst river at dead-end road to Leang Londron; 4°51.929'S, 119°37.702'E | HN | - |  |  |  |  |
|  | 192182 | South Sulawesi: Maros carst: River at road Maros - Camba, in Battunuang; 5°3.083'S, 119°43.109'E | HN | - |  |  |  |  |
|  | 192185 | South Sulawesi: Maros carst: Stream N of road Maros - Camba; 5°2.569'S, 119°42.219'E | HN | - |  |  |  |  |
| *wesseli* | 190430 | South Sulawesi: Tominanga River: c. 4.5 km N of mouth at Lake Towuti; 2°37,79'S, 121°31,81'E | KJ850904 | KJ850744 |  |  |  |  |
| *wolterecki* | 190463 | South Sulawesi: Lake Mahalona: S shore, c. 50m W of Tominanga River outflow; 2°36,86'S, 121°30,9'E | KJ850905 | KJ850745 | E | C | C |  |
| *zeamais* | 190482a | South Sulawesi: Lake Matano: N shore, E of Nuha; 2°27,342'S, 121°21,624'E | KJ850906 | KJ850746 |  |  |  |  |
