# Supplementary material for: A Snail Perspective on the Biogeography of Sulawesi, Indonesia: Origin and Intra-Island Dispersal of the Viviparous Freshwater Gastropod Tylomelania
Source: PLoS One. 2014 Jun 27;9(6):e98917. doi: 10.1371/journal.pone.0098917 (PMC4090239; doi:10.1371/journal.pone.0098917)
Supplement: Table S2 — Haplotypes of Tylomelania (see Figures S3 & S4) and their assignment to species and museum vouchers. The numbers listed under ZMB Moll. are the museum accession numbers of the malacological collection of the Museum für Naturkunde Berlin and provide a link to the sample locality information in Table S1. GenBank accession numbers (for each haplotype) are for the specimen indicated in brackets. (DOC) [file pone.0098917.s006.doc]

Table S2. Haplotypes of *Tylomelania* (see Figures S3 & S4) and their assignment to species and museum vouchers. The numbers listed under ZMB Moll. are the museum accession numbers of the malacological collection of the Museum für Naturkunde Berlin and provide a link to the sample locality information in Table S1. GenBank accession numbers (for each haplotype) are for the specimen indicated in brackets.

| **Haplotype** | **N** | **Species** | **ZMB Moll.** | **Individuals** | **GenBank accession no.** |
| --- | --- | --- | --- | --- | --- |
| 1 | 45 | *Tylomelania* spec. 23 | 190788 | 1-6,8 | KJ850886 (190788.1) |
|  |  | *Tylomelania* spec. 23 | 190791 | 1-3 |  |
|  |  | *Tylomelania* spec. 23 | 192162 | 1-6 |  |
|  |  | *Tylomelania* spec. 8 | 190682 | 1-8 |  |
|  |  | *Tylomelania* spec. 8 | 190683b | 1 |  |
|  |  | *Tylomelania* spec. 8 | 190683d | 3 |  |
|  |  | *Tylomelania* spec. 8 | 190683g | 1-3 |  |
|  |  | *Tylomelania* spec. 8 | 190790 | 5 |  |
|  |  | *Tylomelania robusta* | 192154 | 3-5 |  |
|  |  | *Tylomelania wallacei* | 115516 | 4 |  |
|  |  | *Tylomelania wallacei* | 190685a | 2,4 |  |
|  |  | *Tylomelania wallacei* | 190685b | 2,3 |  |
|  |  | *Tylomelania wallacei* | 190685c | 3,4 |  |
|  |  | *Tylomelania wallacei* | 190789 | 3 |  |
|  |  | *Tylomelania wallacei* | 190792 | 1 |  |
|  |  | *Tylomelania wallacei* | 190795 | 3 |  |
|  |  | *Tylomelania wallacei* | 192163 | 2,4 |  |
| 2 | 19 | *Tylomelania* spec. 23 | 192149 | 1,2,5-8 | KJ933712 (192149.1) |
|  |  | *Tylomelania* spec. 23 | 192173 | 1-3 |  |
|  |  | *Tylomelania* spec. 8 | 192171a | 1 |  |
|  |  | *Tylomelania* spec. 8 | 192171b | 1-4 |  |
|  |  | *Tylomelania wallacei* | 192150 | 1-3,8 |  |
|  |  | *Tylomelania wallacei* | 192172b | 1 |  |
| 3 | 4 | *Tylomelania* spec. 23 | 192149 | 3,4 | KJ933713 (192149.3) |
|  |  | *Tylomelania* spec. 23 | 192173 | 5,6 |  |
| 4 | 1 | *Tylomelania* spec. 23 | 192173 | 4 | KJ933714 (192173.4) |
| 5 | 14 | *Tylomelania celebicola* | 190529 | 1,2,4-7 | KJ850750 (190529.1) |
|  |  | *Tylomelania celebicola* | 190531 | 1,2 |  |
|  |  | *Tylomelania celebicola* | 191609 | 1 |  |
|  |  | *Tylomelania* spec. 13 | 191093 | 1,3,4 |  |
|  |  | *Tylomelania celebicola* | 191094b | 2 |  |
|  |  | *Tylomelania* spec. 13 | 191120 | 8 |  |
| 6 | 1 | *Tylomelania celebicola* | 190529 | 3 | KJ933715 (190529.3) |
| 7 | 1 | *Tylomelania celebicola* | 190529 | 8 | KJ933716 (190529.8) |
| 8 | 5 | *Tylomelania celebicola* | 190530 | 2 |  |
|  |  | *Tylomelania celebicola* | 190532 | 1-4 | KJ850752 (190532.2) |
| 9 | 19 | *Tylomelania celebicola* | 190531 | 3 | KJ933717 (190531.3) |
|  |  | *Tylomelania celebicola* | 191607 | 1-5,7 |  |
|  |  | *Tylomelania celebicola* | 191608a | 1-6 |  |
|  |  | *Tylomelania celebicola* | 191608b | 1-5 |  |
|  |  | *Tylomelania celebicola* | 191608c | 1 |  |
| 10 | 1 | *Tylomelania celebicola* | 191109 | 1 | KJ933718 (191109.1) |
| 11 | 1 | *Tylomelania celebicola* | 191109 | 2 | KJ933719 (191109.2) |
| 12 | 7 | *Tylomelania celebicola* | 191110 | 1 | KJ933720 (191110.1) |
|  |  | *Tylomelania* spec. 13 | 191120 | 2,6 |  |
|  |  | *Tylomelania* spec. 13 | 191611a | 2 |  |
|  |  | *Tylomelania* spec. 13 | 191611b | 1 |  |
|  |  | *Tylomelania* spec. 13 | 191613b | 1,2 |  |
| 13 | 3 | *Tylomelania celebicola* | 191110 | 2,4,5 | KJ933721 (191110.2) |
| 14 | 10 | *Tylomelania celebicola* | 191110 | 3 | KJ933722 (191110.3) |
|  |  | *Tylomelania celebicola* | 191610 | 1-4,7-11 |  |
| 15 | 1 | *Tylomelania celebicola* | 191607 | 6 | KJ933723 (191607.6) |
| 16 | 9 | *Tylomelania celebicola* | 191610 | 5 | KJ933724 (191610.5) |
|  |  | *Tylomelania* spec. 13 | 191092b | 1 |  |
|  |  | *Tylomelania* spec. 13 | 191116a | 2,3 |  |
|  |  | *Tylomelania* spec. 13 | 191116b | 1 |  |
|  |  | *Tylomelania* spec. 13 | 191118b | 1,3,6 |  |
|  |  | *Tylomelania* spec. 13 | 191611b | 3 |  |
| 17 | 4 | *Tylomelania celebicola* | 191610 | 6 | KJ933725 (191610.6) |
|  |  | *Tylomelania* spec. 13 | 191118a | 1,4 |  |
|  |  | *Tylomelania* spec. 13 | 191118b | 2 |  |
| 18 | 22 | *Tylomelania carota* | 190082 | 1-6 | AY311958 (190082.1) |
|  |  | *Tylomelania carota* | 190193 | 1,3-5 |  |
|  |  | *Tylomelania* spec. 3 | 191018 | 2 |  |
|  |  | *Tylomelania* spec. 3 | 191019 | 2-6,8 |  |
|  |  | *Tylomelania* spec. 3 | 191020b | 1 |  |
|  |  | *Tylomelania perconica* | 190822 | 2,3,5,6 |  |
| 19 | 1 | *Tylomelania carota* | 190193 | 2 | AY311957 (190193.2) |
| 20 | 4 | *Tylomelania helmuti* | 190209 | 21 |  |
|  |  | *Tylomelania helmuti* | 190510 | 1,2 | EF140851 (190510.1) |
|  |  | *Tylomelania helmuti* | 190699 | 3 |  |
| 21 | 2 | *Tylomelania helmuti* | 190510 | 3,4 | KJ933726 (190510.3) |
| 22 | 14 | *Tylomelania helmuti* | 190515 | 1-12 | EF140852 (190515.2) |
|  |  | *Tylomelania helmuti* | 190516 | 2,3 |  |
| 23 | 11 | *Tylomelania helmuti* | 190516 | 1 | EF140853 (190516.1) |
|  |  | *Tylomelania helmuti* | 190748a | 1,2,4-7 |  |
|  |  | *Tylomelania helmuti* | 190748b | 1,3 |  |
|  |  | *Tylomelania helmuti* | 190748c | 1,2 |  |
| 24 | 3 | *Tylomelania helmuti* | 190525 | 2 | KJ933727 (190525.2) |
|  |  | *Tylomelania helmuti* | 190526 | 4,5 |  |
| 25 | 10 | *Tylomelania helmuti* | 190525 | 6 |  |
|  |  | *Tylomelania helmuti* | 190526 | 1 | EF140854 (190526.1) |
|  |  | *Tylomelania helmuti* | 190528 | 1,2 |  |
|  |  | *Tylomelania helmuti* | 190746 | 4 |  |
|  |  | *Tylomelania helmuti* | 190747 | 2,4-7 |  |
| 26 | 10 | *Tylomelania helmuti* | 190527 | 1-6 | EF140855 (190527.6) |
|  |  | *Tylomelania helmuti* | 190528 | 3-6 |  |
| 27 | 4 | *Tylomelania helmuti* | 190699 | 1,2 | KJ850756 (190699.2) |
|  |  | *Tylomelania* spec. 3 | 190692 | 5 |  |
|  |  | *Tylomelania* spec. 3 | 190698a | 6 |  |
| 28 | 7 | *Tylomelania helmuti* | 190700 | 1,3-8 | KJ850758 (190700.4) |
| 29 | 12 | *Tylomelania helmuti* | 190701a | 1,3,5-10,12,13 |  |
|  |  | *Tylomelania helmuti* | 190701b | 1,2 | EF140856 (190701b.1) |
| 30 | 2 | *Tylomelania helmuti* | 190701a | 2,11 | KJ933728 (190701a.2) |
| 31 | 1 | *Tylomelania helmuti* | 190701a | 4 | KJ933729 (190701a.4) |
| 32 | 1 | *Tylomelania helmuti* | 190746 | 1 | KJ933730 (190746.1) |
| 33 | 3 | *Tylomelania helmuti* | 190746 | 2,3,5 | KJ850760 (190746.5) |
| 34 | 1 | *Tylomelania helmuti* | 190747 | 1 | KJ933731 (190747.1) |
| 35 | 1 | *Tylomelania helmuti* | 190747 | 3 | KJ933732 (190747.3) |
| 36 | 2 | *Tylomelania helmuti* | 190748a | 3 | KJ850762 (190748a.3) |
|  |  | *Tylomelania helmuti* | 190748b | 2 |  |
| 37 | 8 | *Tylomelania helmuti* | 190749a | 1-6 | KJ850765 (190749a.2) |
|  |  | *Tylomelania helmuti* | 190749b | 1,2 |  |
| 38 | 3 | *Tylomelania helmuti* | 191026 | 1,3,5 | KJ933733 (191026.1) |
| 39 | 1 | *Tylomelania helmuti* | 191026 | 2 | KJ933734 (191026.2) |
| 40 | 19 | *Tylomelania helmuti* | 191026 | 4 | KJ850766 (191026.4) |
|  |  | *Tylomelania* spec. 1 | 190750 | 1-6 |  |
|  |  | *Tylomelania* spec. 1 | 191027a | 1-4,6,7 |  |
|  |  | *Tylomelania* spec. 1 | 191027b | 1-6 |  |
| 41 | 1 | *Tylomelania helmuti* | 191026 | 6 | KJ933735 (191026.6) |
| 42 | 3 | *Tylomelania helmuti* | 191028 | 1,4,6 | KJ933736 (191028.1) |
| 43 | 2 | *Tylomelania helmuti* | 191028 | 2,5 | KJ933737 (191028.2) |
| 44 | 1 | *Tylomelania helmuti* | 191028 | 3 | KJ850767 (191028.3) |
| 45 | 14 | *Tylomelania* spec. 2 | 190690 | 1-7,9, 10,12-16 | KJ850798 (190690.2) |
| 46 | 1 | *Tylomelania* spec. 2 | 190690 | 11 | KJ933738 (190690.11) |
| 47 | 1 | *Tylomelania* spec. 2 | 190690 | 8 | KJ933739 (190690.8) |
| 48 | 2 | *Tylomelania* spec. 21 | 191134 | 1,2 | KJ850880 (191134.1) |
| 49 | 4 | *Tylomelania* spec. 21 | 191134 | 3-6 | KJ933740 (191134.3) |
| 50 | 10 | *Tylomelania* spec. 21 | 191135a | 1-10 | KJ850882 (191135a.1) |
| 51 | 4 | *Tylomelania* spec. 24 | 190536 | 1-3,5 | EF140860 (190536.1) |
| 52 | 1 | *Tylomelania* spec. 24 | 190536 | 4 | KJ933741 (190536.4) |
| 53 | 4 | *Tylomelania* spec. 24 | 190536 | 6 |  |
|  |  | *Tylomelania* spec. 24 | 190537 | 1-3 | KJ850889 (190537.3) |
| 54 | 2 | *Tylomelania* spec. 24 | 190537 | 4,5 | KJ933742 (190537.4) |
| 55 | 1 | *Tylomelania* spec. 24 | 190537 | 6 | KJ933743 (190537.6) |
| 56 | 9 | *Tylomelania* spec. 3 | 190512 | 1,3-5 | EF140861 (190512.5) |
|  |  | *Tylomelania* spec. 1 | 190062 | 3,5-8 |  |
| 57 | 11 | *Tylomelania* spec. 3 | 190512 | 2 |  |
|  |  | *Tylomelania* spec. 3 | 190697 | 3 |  |
|  |  | *Tylomelania* spec. 3 | 191018 | 4 | KJ850811 (191018.4) |
|  |  | *Tylomelania* spec. 3 | 191020a | 1 |  |
|  |  | *Tylomelania* spec. 3 | 191020b | 2 |  |
|  |  | *Tylomelania* spec. 3 | 190695 | 1-6 |  |
| 58 | 1 | *Tylomelania* spec. 3 | 190512 | 6 | KJ933744 (190512.6) |
| 59 | 17 | *Tylomelania* spec. 3 | 190513 | 1,3,4 | KJ850802 (190513.3) |
|  |  | *Tylomelania* spec. 3 | 190514 | 1 |  |
|  |  | *Tylomelania* spec. 3 | 190691 | 1-6 |  |
|  |  | *Tylomelania* spec. 3 | 190696 | 2,9 |  |
|  |  | *Tylomelania* spec. 3 | 190698a | 1,3,7 |  |
|  |  | *Tylomelania* spec. 3 | 190761 | 3,6 |  |
| 60 | 11 | *Tylomelania* spec. 3 | 190513 | 2 |  |
|  |  | *Tylomelania* spec. 3 | 190692 | 4 |  |
|  |  | *Tylomelania* spec. 3 | 190696 | 1,4,7 |  |
|  |  | *Tylomelania* spec. 3 | 190761 | 1,2,4 | EF140863 (190761.2) |
|  |  | *Tylomelania* spec. 3 | 191024 | 1-3 |  |
| 61 | 20 | *Tylomelania* spec. 3 | 190514 | 2,6 | KJ850803 (190514.2) |
|  |  | *Tylomelania* spec. 3 | 190692 | 3 |  |
|  |  | *Tylomelania* spec. 3 | 190696 | 5 |  |
|  |  | *Tylomelania* spec. 3 | 190697 | 1,4,5 |  |
|  |  | *Tylomelania* spec. 3 | 190698a | 2,4,5,8 |  |
|  |  | *Tylomelania* spec. 3 | 190698b | 1,2 |  |
|  |  | *Tylomelania* spec. 3 | 191119 | 1,2,4,5 |  |
| 62 | 3 | *Tylomelania* spec. 3 | 190681 | 1,2,6 | KJ933745 (190681.1) |
| 63 | 3 | *Tylomelania* spec. 3 | 190681 | 3-5 | KJ850804 (190681.3) |
| 64 | 2 | *Tylomelania* spec. 3 | 190681 | 7,8 | KJ933746 (190681.7) |
| 65 | 2 | *Tylomelania* spec. 3 | 190692 | 1 | KJ933747 (190692.1) |
|  |  | *Tylomelania* spec. 3 | 190697 | 2 |  |
| 66 | 1 | *Tylomelania* spec. 3 | 190692 | 2 | KJ933748 (190692.2) |
| 67 | 1 | *Tylomelania* spec. 3 | 190696 | 3 | KJ933749 (190696.3) |
| 68 | 5 | *Tylomelania* spec. 3 | 190696 | 6 | KJ933750 (190696.6) |
|  |  | *Tylomelania* spec. 3 | 191018 | 1,3,5,6 |  |
| 69 | 1 | *Tylomelania* spec. 3 | 190696 | 8 | KJ933751 (190696.8) |
| 70 | 1 | *Tylomelania* spec. 3 | 190761 | 5 | KJ933752 (190761.5) |
| 71 | 2 | *Tylomelania* spec. 3 | 191019 | 1,7 | KJ850812 (191019.7) |
| 72 | 1 | *Tylomelania* spec. 3 | 191020a | 2 | KJ850813 (191020a.2) |
| 73 | 2 | *Tylomelania* spec. 3 | 191020a | 3,4 | KJ933753 (191020a.3) |
| 74 | 8 | *Tylomelania* spec. 3 | 191025 | 1-6 | KJ850816 (191025.4) |
|  |  | *Tylomelania* spec. 3 | 191606 | 1,2 |  |
| 75 | 1 | *Tylomelania* spec. 3 | 191119 | 3 | KJ933754 (191119.3) |
| 76 | 7 | *Tylomelania perconica* | 190083 | 6-8,10 | KJ933755 (190083.6) |
|  |  | *Tylomelania* spec. 4 | 190844 | 1 |  |
|  |  | *Tylomelania* sp. | 193899 | 1,2 |  |
| 77 | 1 | *Tylomelania perconica* | 190083 | 5 | AY312001 (190083.5) |
| 78 | 10 | *Tylomelania perconica* | 190083 | 9 |  |
|  |  | *Tylomelania* spec. 4 | 190191 | 107-111 |  |
|  |  | *Tylomelania* spec. 4 | 190192 | 3,5 |  |
|  |  | *Tylomelania* spec. 4 | 190857 | 2 | KJ850820 (190857.2) |
|  |  | *Tylomelania* spec. 4 | 192134 | 8 |  |
| 79 | 2 | *Tylomelania perconica* | 190822 | 1,4 | KJ933756 (190822.1) |
| 80 | 5 | *Tylomelania perfecta* | 190797 | 1-3,6 | KJ850780 (190797.3) |
|  |  | *Tylomelania wallacei* | 192182 | 1 |  |
| 81 | 2 | *Tylomelania perfecta* | 190797 | 4,5 | KJ850781 (190797.4) |
| 82 | 26 | *Tylomelania perfecta* | 191639 | 1 | KJ850782 (191639.1) |
|  |  | *Tylomelania* spec. 8 | 192146 | 1-5 |  |
|  |  | *Tylomelania robusta* | 192152 | 1-6 |  |
|  |  | *Tylomelania* spec. 18 | 191143 | 1-8 |  |
|  |  | *Tylomelania* spec. 18 | 192177 | 1-6 |  |
| 83 | 45 | *Tylomelania perfecta* | 191639 | 2 | KJ850783 (191639.2) |
|  |  | *Tylomelania* spec. 8 | 190683d | 1 |  |
|  |  | *Tylomelania* spec. 8 | 190688b | 1,3 |  |
|  |  | *Tylomelania* spec. 8 | 190790 | 1-4,6 |  |
|  |  | *Tylomelania* spec. 8 | 190793 | 1-6 |  |
|  |  | *Tylomelania* spec. 8 | 190810 | 1,2,4-8 |  |
|  |  | *Tylomelania* spec. 8 | 191144a | 1-4,6 |  |
|  |  | *Tylomelania* spec. 8 | 191144b | 1-6 |  |
|  |  | *Tylomelania* spec. 8 | 191146 | 2,3,6 |  |
|  |  | *Tylomelania robusta* | 192154 | 2,6 |  |
|  |  | *Tylomelania* sp. | 193900 | 1,2 |  |
|  |  | *Tylomelania* sp. | 193901 | 1 |  |
|  |  | *Tylomelania wallacei* | 190789 | 1,2 |  |
|  |  | *Tylomelania wallacei* | 190792 | 3 |  |
|  |  | *Tylomelania wallacei* | 192164 | 1 |  |
| 84 | 3 | *Tylomelania* spec. 4 | 190191 | 106 | KJ933757 (190191.106) |
|  |  | *Tylomelania* spec. 4 | 192134 | 7 |  |
|  |  | *Tylomelania* sp. | 193902 | 1 |  |
| 85 | 1 | *Tylomelania* spec. 4 | 190192 | 1 | AY312000 (190192.1) |
| 86 | 4 | *Tylomelania* spec. 4 | 190192 | 4 |  |
|  |  | *Tylomelania* spec. 4 | 190844 | 4-6 | KJ850819 (190844.5) |
| 87 | 1 | *Tylomelania* spec. 4 | 190192 | 6 | KJ933758 (190192.7) |
| 88 | 1 | *Tylomelania* spec. 4 | 190192 | 7 | KJ933759 (190192.7) |
| 89 | 48 | *Tylomelania* spec. 4 | 190831 | 1,3 |  |
|  |  | *Tylomelania* spec. 4 | 190844 | 2,3 |  |
|  |  | *Tylomelania robusta* | 190811 | 1 |  |
|  |  | *Tylomelania* spec. 14 | 190084 | 1,2,4 | AY312017 (190084.2) |
|  |  | *Tylomelania* spec. 14 | 190189 | 3-8 |  |
|  |  | *Tylomelania* spec. 14 | 191593a | 1-8 |  |
|  |  | *Tylomelania* spec. 14 | 191593b | 1,2,4,5 |  |
|  |  | *Tylomelania* spec. 14 | 191594 | 1 |  |
|  |  | *Tylomelania* spec. 14 | 191596 | 1-6 |  |
|  |  | *Tylomelania* spec. 14 | 190851 | 1,5-8 |  |
|  |  | *Tylomelania* spec. 14 | 190840 | 1-6 |  |
|  |  | *Tylomelania* spec. 14 | 190868 | 1-4 |  |
| 90 | 1 | *Tylomelania* spec. 4 | 190831 | 2 | KJ933760 (190831.2) |
| 91 | 1 | *Tylomelania* spec. 4 | 190831 | 4 | KJ933761 (190831.4) |
| 92 | 1 | *Tylomelania* spec. 4 | 190831 | 5 | KJ933762 (190831.5) |
| 93 | 1 | *Tylomelania* spec. 4 | 190831 | 6 | KJ933763 (190831.6) |
| 94 | 1 | *Tylomelania* spec. 4 | 190831 | 7 | KJ850818 (190831.7) |
| 95 | 1 | *Tylomelania* spec. 4 | 190831 | 8 | KJ933764 (190831.8) |
| 96 | 10 | *Tylomelania* spec. 4 | 190857 | 1,4-6 | KJ933765 (190857.1) |
|  |  | *Tylomelania* spec. 4 | 192134 | 3-6 |  |
|  |  | *Tylomelania* spec. 4 | 193902 | 2,4 |  |
| 97 | 1 | *Tylomelania* spec. 4 | 190857 | 3 | KJ850821 (190857.3) |
| 98 | 1 | *Tylomelania* spec. 4 | 190864 | 1 | KJ850822 (190864.1) |
| 99 | 2 | *Tylomelania* spec. 4 | 190864 | 2,5 | KJ933766 (190864.2) |
| 100 | 3 | *Tylomelania* spec. 4 | 190864 | 3,4,6 | KJ933767 (190864.3) |
| 101 | 3 | *Tylomelania* spec. 4 | 190867 | 1-3 | KJ850823 (190867.1) |
| 102 | 1 | *Tylomelania* spec. 4 | 190867 | 4 | KJ933768 (190867.4) |
| 103 | 2 | *Tylomelania* spec. 4 | 192134 | 1 | KJ933769 (192134.1) |
|  |  | *Tylomelania* spec. 4 | 193902 | 3 |  |
| 104 | 1 | *Tylomelania* spec. 4 | 192134 | 2 | KJ933770 (192134.2) |
| 105 | 12 | *Tylomelania* spec. 5 | 190809 | 1-6 | KJ850824 (190809.5) |
|  |  | *Tylomelania* spec. 5 | 190841 | 1-6 |  |
| 106 | 4 | *Tylomelania* spec. 5 | 190850 | 2,5-7 | KJ850826 (190850.5) |
| 107 | 5 | *Tylomelania* spec. 5 | 191136 | 1-3 | KJ850828 (191136.2) |
|  |  | *Tylomelania* spec. 5 | 191137 | 2,8 |  |
| 108 | 6 | *Tylomelania* spec. 5 | 191137 | 1,3-7 | KJ850829 (191137.1) |
| 109 | 3 | *Tylomelania* spec. 8 | 115517 | 1,2 | KJ933771 (115517.1) |
|  |  | *Tylomelania wallacei* | 115516 | 1 |  |
| 110 | 12 | *Tylomelania* spec. 8 | 190683b | 2 |  |
|  |  | *Tylomelania* spec. 8 | 190794 | 1-3 | KJ850843 (190794.3) |
|  |  | *Tylomelania wallacei* | 190683a | 1 |  |
|  |  | *Tylomelania wallacei* | 190683f | 2 |  |
|  |  | *Tylomelania wallacei* | 190685c | 2 |  |
|  |  | *Tylomelania wallacei* | 190795 | 4 |  |
|  |  | *Tylomelania wallacei* | 192150 | 6 |  |
|  |  | *Tylomelania wallacei* | 192167a | 6 |  |
|  |  | *Tylomelania wallacei* | 192172a | 3,6 |  |
| 111 | 9 | *Tylomelania* spec. 8 | 190687 | 1-3,5,8-12 | KJ850833 (190687.2) |
| 112 | 11 | *Tylomelania* spec. 8 | 190687 | 4,6,7 |  |
|  |  | *Tylomelania* spec. 8 | 190689 | 1-8 | KJ850839 (190689.7) |
| 113 | 1 | *Tylomelania* spec. 8 | 190688a | 10 | KJ933772 (190688a.10) |
| 114 | 1 | *Tylomelania* spec. 8 | 190688a | 11 | KJ933773 (190688a.11) |
| 115 | 8 | *Tylomelania* spec. 8 | 190688a | 1,3,5,7-9 | KJ850834 (190688a.3) |
|  |  | *Tylomelania* spec. 8 | 190688b | 2,4 |  |
| 116 | 3 | *Tylomelania* spec. 8 | 190688a | 2,4,6 | KJ850835 (190688a.6) |
| 117 | 4 | *Tylomelania* spec. 8 | 190796 | 1-4 | KJ933774 (190796.1) |
| 118 | 5 | *Tylomelania* spec. 8 | 190805 | 1-5 | KJ850844 (190805.2) |
| 119 | 5 | *Tylomelania* spec. 8 | 190806 | 1,3-6 | KJ850845 (190806.1) |
| 120 | 9 | *Tylomelania* spec. 8 | 190806 | 2 | KJ850846 (190806.2) |
|  |  | *Tylomelania* spec. 8 | 190807 | 1-4 |  |
|  |  | *Tylomelania robusta* | 192155 | 1-4 |  |
| 121 | 21 | *Tylomelania* spec. 8 | 190815a | 1-6 | KJ850849 (190815a.2) |
|  |  | *Tylomelania* spec. 8 | 190815b | 2,4,5,7,8 |  |
|  |  | *Tylomelania* spec. 8 | 190855 | 1-4,6 |  |
|  |  | *Tylomelania* spec. 8 | 191145 | 1,3-6 |  |
| 122 | 4 | *Tylomelania* spec. 8 | 190815b | 1,3,6 | KJ850852 (190815b.6) |
|  |  | *Tylomelania* spec. 8 | 191145 | 2 |  |
| 123 | 2 | *Tylomelania* spec. 8 | 190855 | 5 |  |
|  |  | *Tylomelania* spec. 8 | 190866 | 5 | KJ850858 (190866.5) |
| 124 | 19 | *Tylomelania* spec. 8 | 190856 | 1-4 | KJ850854 (190856.2) |
|  |  | *Tylomelania* spec. 8 | 190862 | 1-6 |  |
|  |  | *Tylomelania robusta* | 190811 | 3,4 |  |
|  |  | *Tylomelania robusta* | 190845 | 1-6 |  |
|  |  | *Tylomelania* sp. | 193903 | 1 |  |
| 125 | 5 | *Tylomelania* spec. 8 | 190866 | 1-3,6,8 | KJ850857 (190866.1) |
| 126 | 1 | *Tylomelania* spec. 8 | 190866 | 4 | KJ933775 (190866.4) |
| 127 | 1 | *Tylomelania* spec. 8 | 190866 | 7 | KJ933776 (190866.7) |
| 128 | 2 | *Tylomelania* spec. 8 | 190869 | 1,2 | KJ850859 (190869.1) |
| 129 | 1 | *Tylomelania* spec. 8 | 191144a | 5 | KJ933777 (191144a.5) |
| 130 | 3 | *Tylomelania* spec. 8 | 192183 | 1 | KJ933778 (192183.1) |
|  |  | *Tylomelania* spec. 8 | 192188 | 2 |  |
|  |  | *Tylomelania wallacei* | 192182 | 3 |  |
| 131 | 1 | *Tylomelania* spec. 8 | 192188 | 1 | KJ933779 (192188.1) |
| 132 | 2 | *Tylomelania robusta* | 190811 | 2 | KJ933780 (190811.2) |
|  |  | *Tylomelania wallacei* | 190685c | 6 |  |
| 133 | 2 | *Tylomelania robusta* | 190853 | 1,4 | KJ850786 (190853.4) |
| 134 | 3 | *Tylomelania robusta* | 190853 | 2,3,5 | KJ933781 (190853.2) |
| 135 | 8 | *Tylomelania robusta* | 190865 | 1-8 | KJ850787 (190865.2) |
| 136 | 2 | *Tylomelania robusta* | 192154 | 1 | KJ850789 (192154.1) |
|  |  | *Tylomelania wallacei* | 192148 | 1 |  |
| 137 | 1 | *Tylomelania scalariopsis* | 190009 | 4 | AY311962 (190009.4) |
| 138 | 16 | *Tylomelania scalariopsis* | 190859 | 1 | KJ850792 (190859.1) |
|  |  | *Tylomelania* spec. 20 | 190832 | 2 |  |
|  |  | *Tylomelania* spec. 20 | 191597 | 1-6 |  |
|  |  | *Tylomelania* spec. 20. | 191598 | 1,3,6-8 |  |
|  |  | *Tylomelania* spec. 20 | 191599 | 2,3,7 |  |
| 139 | 4 | *Tylomelania scalariopsis* | 190863 | 4 | KJ850793 (190863.4) |
|  |  | *Tylomelania scalariopsis* | 190008 | 3 |  |
|  |  | *Tylomelania* spec. 20 | 190207 | 1 |  |
|  |  | *Tylomelania* spec. 20 | 191605 | 11 |  |
| 140 | 8 | *Tylomelania scalariopsis* | 191603 | 1 | KJ933782 (191603.1) |
|  |  | *Tylomelania* spec. 20 | 190832 | 3 |  |
|  |  | *Tylomelania* spec. 20 | 191598 | 2,4,5 |  |
|  |  | *Tylomelania* spec. 20 | 191599 | 1,5,8 |  |
| 141 | 3 | *Tylomelania scalariopsis* | 191603 | 2,3 | KJ933783 (191603.2) |
|  |  | *Tylomelania* spec. 20 | 191604 | 2 |  |
| 142 | 3 | *Tylomelania scalariopsis* | 191603 | 4 | KJ933784 (191603.4) |
|  |  | *Tylomelania* spec. 20 | 191604 | 1,3 |  |
| 143 | 1 | *Tylomelania* spec. 1 | 191027a | 5 | KJ850797 (191027a.5) |
| 144 | 1 | *Tylomelania* spec. 17 | 190812 | 2 | KJ933785 (190812.2) |
| 145 | 7 | *Tylomelania* spec. 17 | 190812 | 3 | KJ850870 (190812.3) |
|  |  | *Tylomelania* spec. 20 | 191600 | 1-6 |  |
| 146 | 4 | *Tylomelania* spec. 18 | 191131 | 1,2 | KJ933786 (191131.1) |
|  |  | *Tylomelania* spec. 18 | 191133 | 7,10 |  |
| 147 | 10 | *Tylomelania* spec. 18 | 191131 | 3 | KJ850872 (191131.3) |
|  |  | *Tylomelania* spec. 26 | 195096 | 14,21 |  |
|  |  | *Tylomelania wallacei* | 115515 | 2,6 |  |
|  |  | *Tylomelania wallacei* | 190683a | 2 |  |
|  |  | *Tylomelania wallacei* | 192185 | 1,4,6,8 |  |
| 148 | 8 | *Tylomelania* spec. 18 | 191132 | 1-8 | KJ933787 (191132.1) |
| 149 | 8 | *Tylomelania* spec. 18 | 191133 | 1-6,8,9 | KJ850873 (191133.1) |
| 150 | 31 | *Tylomelania* spec. 25 | 190032 | 1 | KJ933788 (190032.1) |
|  |  | *Tylomelania* spec. 25 | 191113 | 5,6 |  |
|  |  | *Tylomelania* spec. 25 | 191612 | 1,3,6 |  |
|  |  | *Tylomelania* spec. 13 | 191092a | 1-4 |  |
|  |  | *Tylomelania* spec. 13 | 191092b | 2 |  |
|  |  | *Tylomelania* spec. 13 | 191092c | 1,3-5 |  |
|  |  | *Tylomelania* spec. 13 | 191093 | 5 |  |
|  |  | *Tylomelania* spec. 13 | 191112a | 3,5,6 |  |
|  |  | *Tylomelania* spec. 13 | 191112b | 1,3,4 |  |
|  |  | *Tylomelania* spec. 13 | 191118a | 2,3,5,6 |  |
|  |  | *Tylomelania* spec. 13 | 191118b | 4,5 |  |
|  |  | *Tylomelania* spec. 13 | 191613a | 1-3 |  |
| 151 | 4 | *Tylomelania* spec. 25 | 191113 | 1,3,4 | KJ933789 (191113.1) |
|  |  | *Tylomelania* spec. 25 | 191612 | 2 |  |
| 152 | 1 | *Tylomelania* spec. 25 | 191113 | 2 | KJ933790 (191113.2) |
| 153 | 5 | *Tylomelania* spec. 22 | 192189 | 1-4,6 | KJ850884 (192189.1) |
| 154 | 1 | *Tylomelania* spec. 22 | 192189 | 5 | KJ933791 (192189.5) |
| 155 | 1 | *Tylomelania* spec. 13 | 190035 | 1 | AY312013 (190035.1) |
| 156 | 4 | *Tylomelania* spec. 13 | 190035 | 2,3,5,6 | KJ933792 (190035.2) |
| 157 | 1 | *Tylomelania* spec. 13 | 190035 | 4 | KJ933793 (190035.4) |
| 158 | 6 | *Tylomelania* spec. 14 | 190084 | 3 | KJ933794 (1900843) |
|  |  | *Tylomelania* spec. 14 | 191594 | 2 |  |
|  |  | *Tylomelania* spec. 14 | 191595 | 1-3 |  |
|  |  | *Tylomelania* spec. 14 | 190851 | 3 |  |
| 159 | 30 | *Tylomelania* spec. 15 | 190187 | 3,4,6,7 |  |
|  |  | *Tylomelania* spec. 15 | 190188 | 3-8 | AY312004 (190188.3) |
|  |  | *Tylomelania* spec. 15 | 191138 | 3,5 |  |
|  |  | *Tylomelania* spec. 15 | 191139 | 1-8 |  |
|  |  | *Tylomelania* spec. 15 | 191140 | 1-3,8 |  |
|  |  | *Tylomelania* spec. 15 | 191141a | 3,4,7 |  |
|  |  | *Tylomelania* spec. 15 | 191141b | 1,3,6 |  |
| 160 | 1 | *Tylomelania* spec. 15 | 190187 | 5 | KJ933795 (190187.5) |
| 161 | 4 | *Tylomelania* spec. 14 | 190190 | 1,3,5,7 | AY312016 (190190.1) |
| 162 | 1 | *Tylomelania* spec. 14 | 190190 | 4 | KJ933796 (190190.4) |
| 163 | 5 | *Tylomelania* spec. 16 | 190206 | 1 | AY312009 (190206.1) |
|  |  | *Tylomelania* spec. 20 | 190846 | 2,4-6 |  |
| 164 | 4 | *Tylomelania* spec. 16 | 190206 | 2,3,5,6 | KJ933797 (190206.2) |
| 165 | 1 | *Tylomelania* spec. 16 | 190206 | 4 | KJ933798 (190206.4) |
| 166 | 4 | *Tylomelania* spec. 20 | 190832 | 1,4,6 | KJ933799 (190832.1) |
|  |  | *Tylomelania* spec. 20 | 191599 | 6 |  |
| 167 | 1 | *Tylomelania* spec. 20 | 190832 | 5 | KJ850878 (190832.5) |
| 168 | 1 | *Tylomelania* spec. 20 | 190846 | 1 | KJ933800 (190846.1) |
| 169 | 1 | *Tylomelania* spec. 13 | 191092c | 2 | KJ933801 (191092c.2) |
| 170 | 6 | *Tylomelania* spec. 13 | 191093 | 2,6 | KJ933802 (191093.2) |
|  |  | *Tylomelania* spec. 13 | 191121 | 1,2,4,5 |  |
| 171 | 2 | *Tylomelania celebicola* | 191094a | 1 | KJ933803 (191094a.1) |
|  |  | *Tylomelania celebicola* | 191094b | 1 |  |
| 172 | 7 | *Tylomelania* spec. 13 | 191112a | 1,2,4 | KJ933804 (191112a.1) |
|  |  | *Tylomelania* spec. 13 | 191120 | 1,3 |  |
|  |  | *Tylomelania* spec. 13 | 191611a | 1 |  |
|  |  | *Tylomelania* spec. 13 | 191613a | 4 |  |
| 173 | 1 | *Tylomelania* spec. 13 | 191112b | 2 | KJ933805 (191112b.2) |
| 174 | 9 | *Tylomelania* spec. 13 | 191114 | 1-9 | KJ933806 (191114.1) |
| 175 | 9 | *Tylomelania* spec. 13 | 191115 | 1,3,5,6,10 | KJ933807 (191115.1) |
|  |  | *Tylomelania* spec. 13 | 191116a | 1,4 |  |
|  |  | *Tylomelania* spec. 13 | 191116b | 2 |  |
|  |  | *Tylomelania* spec. 13 | 191611b | 2 |  |
| 176 | 5 | *Tylomelania* spec. 13 | 191115 | 2,4,7,9,11 | KJ933808 (191115.2) |
| 177 | 1 | *Tylomelania* spec. 13 | 191115 | 8 | KJ933809 (191115.8) |
| 178 | 2 | *Tylomelania* spec. 13 | 191120 | 4 | KJ933810 (191120.4) |
|  |  | *Tylomelania* spec. 13 | 191611a | 6 |  |
| 179 | 1 | *Tylomelania* spec. 13 | 191120 | 5 | KJ933811 (191120.5) |
| 180 | 1 | *Tylomelania* spec. 13 | 191120 | 7 | KJ933812 (191120.7) |
| 181 | 4 | *Tylomelania* spec. 15 | 191138 | 1,2,6,8 | KJ933813 (191138.1) |
| 182 | 2 | *Tylomelania* spec. 15 | 191138 | 4,7 | KJ933814 (191138.4) |
| 183 | 3 | *Tylomelania* spec. 15 | 191140 | 4 | KJ933815 (191140.4) |
|  |  | *Tylomelania* spec. 15 | 191141a | 1 |  |
|  |  | *Tylomelania* spec. 15 | 191141b | 2 |  |
| 184 | 7 | *Tylomelania* spec. 15 | 191140 | 5 | KJ933816 (191140.5) |
|  |  | *Tylomelania* spec. 15 | 191141a | 2,5,6,8 |  |
|  |  | *Tylomelania* spec. 15 | 191141b | 4,5 |  |
| 185 | 1 | *Tylomelania* spec. 15 | 191140 | 6 | KJ933817 (191140.6) |
| 186 | 1 | *Tylomelania* spec. 15 | 191140 | 7 | KJ933818 (191140.7) |
| 187 | 8 | *Tylomelania* spec. 12 | 191142 | 1-8 | KJ850865 (191142.8) |
| 188 | 2 | *Tylomelania* spec. 12 | 191143 | 1,4 | KJ933819 (191143.1) |
| 189 | 7 | *Tylomelania* spec. 12 | 191143 | 2,3,5-9 | KJ933820 (191143.2) |
| 190 | 1 | *Tylomelania* spec. 14 | 191593b | 3 | KJ933821 (191593b.3) |
| 191 | 1 | *Tylomelania* spec. 14 | 191595 | 4 | KJ933822 (191595.4) |
| 192 | 1 | *Tylomelania* spec. 20 | 191599 | 4 | KJ933823 (191599.4) |
| 193 | 5 | *Tylomelania* spec. 20 | 191601 | 1,2 | KJ933824 (191601.1) |
|  |  | *Tylomelania* spec. 13 | 192160 | 4,6,8 |  |
| 194 | 1 | *Tylomelania* spec. 20 | 191601 | 3 | KJ933825 (191601.3) |
| 195 | 9 | *Tylomelania* spec. 20 | 191602 | 1,3-10 | KJ933826 (191602.1) |
| 196 | 1 | *Tylomelania* spec. 20 | 191602 | 2 | KJ933827 (191602.2) |
| 197 | 1 | *Tylomelania* spec. 20 | 191605 | 10 | KJ933828 (191605.10) |
| 198 | 1 | *Tylomelania* spec. 20 | 191605 | 12 | KJ933829 (191605.12) |
| 199 | 3 | *Tylomelania* spec. 20 | 191605 | 1,8,9 | KJ933830 (191605.1) |
| 200 | 1 | *Tylomelania* spec. 20 | 191605 | 2 | KJ933831 (191605.2) |
| 201 | 2 | *Tylomelania* spec. 20 | 191605 | 3,5 | KJ933832 (191605.3) |
| 202 | 1 | *Tylomelania* spec. 20 | 191605 | 4 | KJ933833 (191605.4) |
| 203 | 1 | *Tylomelania* spec. 20 | 191605 | 6 | KJ933834 (191605.6) |
| 204 | 1 | *Tylomelania* spec. 20 | 191605 | 7 | KJ933835 (191605.7) |
| 205 | 1 | *Tylomelania* spec. 13 | 192158 | 1 | KJ933836 (192158.1) |
| 206 | 2 | *Tylomelania* spec. 13 | 192158 | 2,3 | KJ933837 (192158.2) |
| 207 | 2 | *Tylomelania* spec. 13 | 192158 | 4,6 | KJ933838 (192158.4) |
| 208 | 3 | *Tylomelania* spec. 13 | 192158 | 5,7,8 | KJ933839 (192158.5) |
| 209 | 4 | *Tylomelania* spec. 13 | 192160 | 1-3,7 | KJ933840 (192160.1) |
| 210 | 1 | *Tylomelania* spec. 13 | 192160 | 5 | KJ933841 (192160.5) |
| 211 | 1 | *Tylomelania* sp. 4 | 193899 | 3 | KJ933842 (193899.3) |
| 212 | 2 | *Tylomelania* spec. 14 | 190851 | 2,4 | KJ933843 (190851.2) |
| 213 | 2 | *Tylomelania* spec. 19 | 190693 | 1,2 | KJ850877 (190693.1) |
| 214 | 3 | *Tylomelania* spec. 19 | 190694 | 1 | EF140865 (190694.1) |
|  |  | *Tylomelania* spec. 19 | 190839 | 2,3 |  |
| 215 | 3 | *Tylomelania wallacei* | 115515 | 1,3,5 | KJ933844 (115515.1) |
| 216 | 1 | *Tylomelania wallacei* | 115515 | 4 | KJ933845 (115515.4) |
| 217 | 1 | *Tylomelania wallacei* | 115516 | 2 | KJ933846 (115516.2) |
| 218 | 4 | *Tylomelania wallacei* | 115516 | 3 | KJ933847 (115516.3) |
|  |  | *Tylomelania wallacei* | 190683a | 4 |  |
|  |  | *Tylomelania wallacei* | 190683c | 3 |  |
|  |  | *Tylomelania wallacei* | 190683e | 1 |  |
| 219 | 9 | *Tylomelania wallacei* | 190211 | 3 |  |
|  |  | *Tylomelania wallacei* | 190798a | 2,4,5 | KJ850902 (190798a.2) |
|  |  | *Tylomelania wallacei* | 190798b | 1-5 |  |
| 220 | 1 | *Tylomelania wallacei* | 190683a | 3 | KJ933848 (190683a.3) |
| 221 | 4 | *Tylomelania wallacei* | 190683c | 1 | KJ933849 (190683c.1) |
|  |  | *Tylomelania wallacei* | 190795 | 2 |  |
|  |  | *Tylomelania wallacei* | 192172a | 5 |  |
|  |  | *Tylomelania wallacei* | 192172b | 2 |  |
| 222 | 1 | *Tylomelania wallacei* | 190683c | 2 | KJ933850 (190683c.2) |
| 223 | 4 | *Tylomelania wallacei* | 190683e | 2 | KJ933851 (190683e.2) |
|  |  | *Tylomelania wallacei* | 190685a | 6 |  |
|  |  | *Tylomelania wallacei* | 190686 | 2 |  |
|  |  | *Tylomelania wallacei* | 192163 | 3 |  |
| 224 | 11 | *Tylomelania wallacei* | 190685a | 1,5 | KJ850893 (190685a.1) |
|  |  | *Tylomelania wallacei* | 190685b | 1 |  |
|  |  | *Tylomelania wallacei* | 190685c | 5 |  |
|  |  | *Tylomelania wallacei* | 190686 | 1 |  |
|  |  | *Tylomelania wallacei* | 192150 | 4,7 |  |
|  |  | *Tylomelania wallacei* | 192163 | 1 |  |
|  |  | *Tylomelania wallacei* | 192167a | 4 |  |
|  |  | *Tylomelania wallacei* | 192172a | 2 |  |
|  |  | *Tylomelania wallacei* | 192172b | 3 |  |
| 225 | 1 | *Tylomelania wallacei* | 190685a | 3 | KJ933852 (190685a.3) |
| 226 | 1 | *Tylomelania wallacei* | 190792 | 2 | KJ933853 (190792.2) |
| 227 | 1 | *Tylomelania wallacei* | 190798b | 6 | KJ933854 (190798b.6) |
| 228 | 1 | *Tylomelania wallacei* | 192167a | 1 | KJ933855 (192167a.1) |
| 229 | 9 | *Tylomelania wallacei* | 192167a | 2,5 | KJ933856 (192167a.2) |
|  |  | *Tylomelania wallacei* | 192167b | 1-4 |  |
|  |  | *Tylomelania wallacei* | 192172a | 1,4 |  |
|  |  | *Tylomelania wallacei* | 192172b | 4 |  |
| 230 | 1 | *Tylomelania wallacei* | 192167a | 3 | KJ933857 (192167a.3) |
| 231 | 1 | *Tylomelania wallacei* | 192182 | 2 | KJ933858 (192182.2) |
| 232 | 1 | *Tylomelania wallacei* | 192185 | 2 | KJ933859 (192185.2) |
| 233 | 3 | *Tylomelania wallacei* | 192185 | 3,5,7 | KJ933860 (192185.3) |
